# The Crayfish Plague Pathogen *Aphanomyces astaci* in Ireland

**DOI:** 10.3390/microorganisms12010102

**Published:** 2024-01-04

**Authors:** Daniel J. Brady, Rossa Meade, Julian D. Reynolds, Andreas Vilcinskas, Kathrin Theissinger

**Affiliations:** 1 Fraunhofer Institute for Molecular Biology and Applied Ecology (IME), Branch for Bioresources, Ohlebergsweg 12, 35392 Gießen, Germany; andreas.vilcinskas@ime.fraunhofer.de; 2 Independent Researcher, Bundoran, Donegal, Ireland; rossameade@gmail.com; 3 Independent Researcher, A94 R8D9 Dublin City, Dublin, Ireland; julian.reynolds11@gmail.com; 4 Institute for Insect Biotechnology, Justus Liebig University Gießen, Heinrich-Buff-Ring 26-32, 35392 Gießen, Germany; kathrin.theissinger@senckenberg.de; 5 LOEWE Centre for Translational Biodiversity Genomics, Senckenberg Biodiversity and Climate Research Centre, Georg-Voigt-Str. 14-16, 60325 Frankfurt Am Main, Germany

**Keywords:** crayfish plague, *Aphanomyces astaci*, *Austropotamobius pallipes*, invasive species, Ireland

## Abstract

Crayfish plague is a devastating disease of European freshwater crayfish and is caused by the oomycete *Aphanomyces astaci* (*Ap. astaci*), believed to have been introduced to Europe around 1860. All European species of freshwater crayfish are susceptible to the disease, including the white-clawed crayfish *Austropotamobius pallipes*. *Ap. astaci* is primarily spread by North American crayfish species and can also disperse rapidly through contaminated wet gear moved between water bodies. This spread, coupled with competition from non-indigenous crayfish, has drastically reduced and fragmented native crayfish populations across Europe. Remarkably, the island of Ireland remained free from the crayfish plague pathogen for over 100 years, providing a refuge for *A. pallipes*. However, this changed in 1987 when a mass mortality event was linked to the pathogen, marking its introduction to the region. Fortunately, crayfish plague was not detected again in Ireland until 2015 when a molecular analysis linked a mass mortality event in the Erne catchment to *Ap. astaci.* Since then, the pathogen has appeared across the island. Between 2015 and 2023, *Ap. astaci* was detected in 18 water catchments, revealing multiple genotypes. Intriguingly, the pathogen in Ireland is present without its natural host species. The uneven distribution of various genetic lineages strongly suggests the human-mediated transport of zoospores via contaminated water equipment as a primary cause of spread. This review details the timeline of these events, *Ap. astaci’s* introduction into Ireland, and its rapid spread. As well, this review references the genotypes that have been determined, and discusses the issue of non-indigenous crayfish species in Ireland and management efforts.

## 1. Introduction

The oomycete *Aphanomyces astaci* (*Ap. astaci*) is an aquatic pathogen responsible for crayfish plague that has devastated freshwater crayfish populations across Europe [1,2]. Native to North America, the pathogen was imported into Europe around 1860, potentially in ballast water to northern Italy [3]. North American NICS usually serve as tolerant vectors of *Ap. astaci* due to host–pathogen co-evolution [2]. However, the indigenous crayfish species of Europe possess low to no innate immune defence against the pathogen [4,5]. To infect a susceptible host, free-swimming microscopic zoospores encyst on the host’s soft cuticle; the pathogen then geminates, and hyphae rapidly develop through the host’s tissues, infiltrating the inner organs and killing the host. Upon death of the host, the asexual pathogen produces sporangia (spore balls), causing a massive sporulation event for the zoospores to find a new host [6]. Local crayfish populations can be lost within weeks [7,8]. Tolerant NICS possess a strong innate immune response against *Ap. astaci* infection and these North American crayfish species repress *Ap. astaci* hyphal growth without killing the parasite, instead acting as mobile vectors of the pathogen [7,9,10]. Since its introduction to Europe, the pathogen has spread across the continent, decimating native European crayfish populations, leading to mortality rates of up to 100% at infected sites [11]. Due to the continuing introduction of alien crayfish into Europe, the crayfish plague pathogen is currently distributed across all of Europe with different genetic lineages of varying virulence [12,13].

The island of Ireland is governed by Irish and Northern Irish authorities in the south and northeast, respectively. The island is low-lying, with large river–lake catchments in the mainly limestone centre, bounded by hilly edges, generally of acid rock [14]. The Irish lowlands are home to a single naturalised species of crayfish, the white-clawed crayfish (*Austropotamobius pallipes* Lereboullet, 1858). As *A. pallipes* is Ireland’s only crayfish species, all other crayfish species are considered NICS in the region. Ireland remained free of crayfish plague and NICS for over 100 years (following European plague outbreaks) with healthy crayfish populations throughout the country and has been referred to as a final stronghold of the species [15].

**Figure 1:**
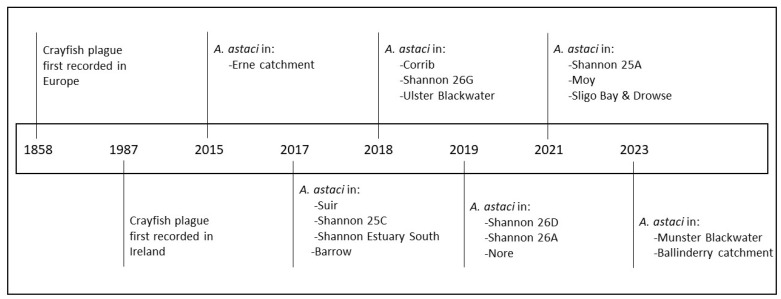
Timeline of the introduction and spread of *Aphanomyces astaci* to Europe and the subsequent introductions and spread across water catchments around the island of Ireland. Codes refer to specific subcatchments within larger catchments.

A temporally isolated crayfish plague mass mortality event occurred in 1987 but was confined to a lake and a nearby pilot crayfish farm in central Ireland [16]. Crayfish plague was not recorded again in Ireland until 2015, when a crayfish mass mortality event on the Erne catchment was confirmed to be caused by *Ap. astaci* (Figure 1) using molecular methods [17,18]. In the subsequent years, crayfish mass mortalities have been reported across Ireland, in numerous waterbodies and catchments, and multiple genetic lineages of the pathogen have been reported (see Section 2.3). Some potential mechanisms of spread in Ireland include (1) spores being shed from infected NICS (live and dead); (2) contaminated wet gear, equipment, vehicles, machinery, or any wet or submerged items being relocated between waterbodies; and (3) ornamental crayfish or their tank water being released into the environment. As no NICS have ever been reported at sites testing positive for *Ap. astaci*, the evidence indicates that the microscopic water mould was introduced into Ireland several times by different introduction pathways.

In response to the continued outbreaks of crayfish plague, the National Crayfish Plague Survey Programme (NCPSP) was established by the National Parks & Wildlife Service (NPWS) and is tasked with overseeing invertebrate protection and conservation in the Republic of Ireland (hereafter Ireland). The NCPSP is conducted by the Marine Institute, which has responsibility over aquatic pathogens in Ireland. Inland Fisheries Ireland is also coordinating efforts to reduce the spread of crayfish plague and have close ties with water users and the angling community. In Northern Ireland, governance lies with the Northern Ireland Environment Agency (NIEA), but they have not established any monitoring programs.

In this review, we detail the introduction and spread of *Ap. astaci* in Ireland, beginning by summarising the isolated 1987 mass mortality event. We then continue from the next detection in 2015, culminating with the NCPSP report in 2021. We present a comprehensive summary of the sampling efforts, highlighting the sites across various regional catchments where *Ap. astaci* was positively identified. Attention is given to the diverse genotypes of *Ap. astaci* detected nationwide. However, those data have not been verified by peer review and are provided as putative results in the supplementary information file. Additionally, we include average cycle threshold (Ct) values and associated errors from environmental DNA (eDNA) sampling in cases where *Ap. astaci* was detected and data are available. We outline and discuss the measures implemented by these institutions, tasked to manage the spread of *Ap. astaci* across all of Ireland to protect native species, habitats, and ecosystems.

## 2. *Aphanomyces astaci* in Ireland

The first incidence of crayfish plague in Ireland occurred in 1987 with no further events recorded until 2015 [16,17]. Since then, outbreaks have been occurring periodically, on an almost annual basis. To date, a total of five reports and articles have been published documenting the known incidences of crayfish plague in Ireland (Table 1).

### 2.1. Historical Incidence of Crayfish Plague in Ireland

**Figure 2:**
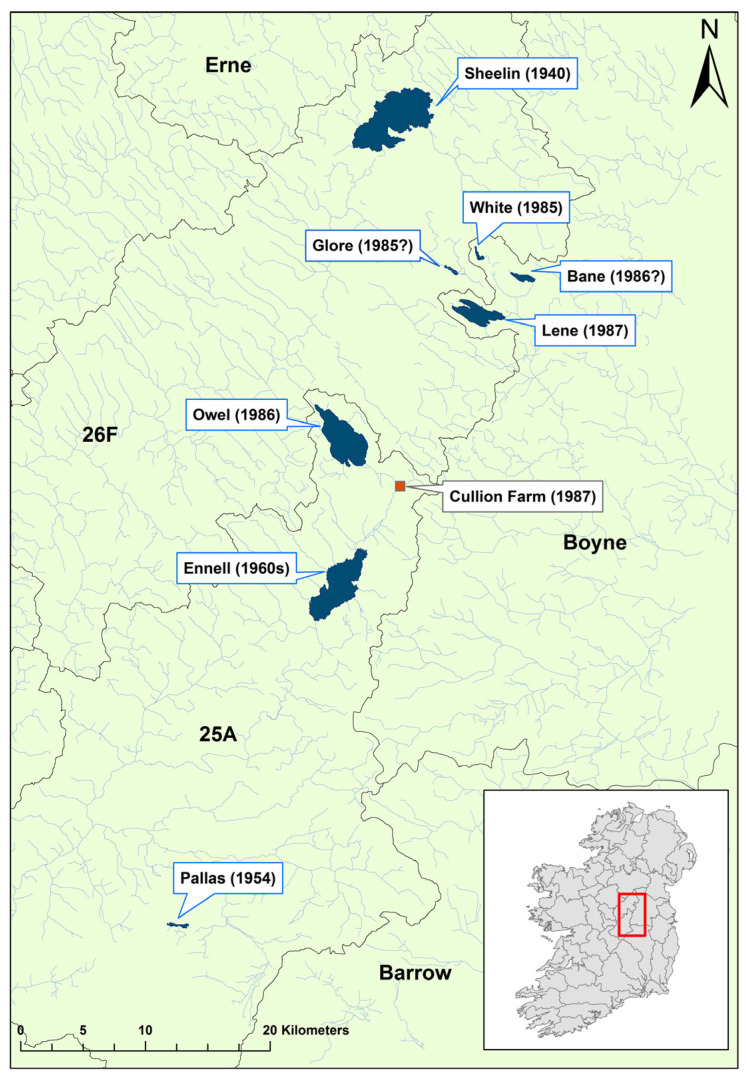
Sites surveyed and affected before and during the 1987 crayfish plague mass mortality event. Map area is contained within red box of the reference map of Ireland. Lakes are labelled with the year that crayfish disappearances were noted preceding 1987 in parentheses, question mark is an estimated year. Erne refers to Erne catchment; Boyne refers to Boyne; Barrow refers to the Barrow; 25A refers to the Lower Shannon (Brosna) 25A; 26F refers to the Lower Shannon 26F catchment. Areas coloured blue represent lakes. Modified from Reynolds, 1988 [16].

White-clawed crayfish populations were historically abundant in most Irish midland lakes [14]. However, before 1987, there had been notable disappearances from several sites, such as Lough Sheelin, Pallas Lake, and Lough Ennell, as well as cyclical disappearances and reappearances in others like Upper River Erne (Figure 2). A 1987 survey of lakes and rivers in central Ireland found that crayfish were absent from White Lake, Lough Glore, and Lough Owel, all of which had previously been known to hold crayfish populations. At the same time, crayfish were still abundant in Lough Lene. In 1987, white-clawed crayfish were harvested from Lough Lene to seed a pilot crayfish farm at Cullion Farm, which indirectly drained into the River Shannon, Ireland’s largest freshwater catchment network. Soon after, crayfish mass mortality events were recorded on both Lough Lene and at the farm. After three weeks, the farm stock was lost entirely, and no surviving crayfish could be found in the lake [16]. Fortunately, the pathogen did not spread down into the Shannon and *Ap. astaci* was not detected on the island again for 35 years.

### 2.2. Recent Cases of Crayfish Plague in Ireland

In 2015, a crayfish mass mortality event on the Bruskey river, County Cavan, was reported to the National Parks and Wildlife Service [17]. In August, a survey conducted by the NPWS removed around 600 dead crayfish from the site and confirmed the animals were infected with *Ap. astaci* (see Section 3.1) [17]. Since then, four surveys have been completed for Irish authorities to monitor the presence and spread of *Ap. astaci* in Ireland. In total, three reports and one peer-reviewed article have been produced by Irish authorities and the researchers who completed the surveys, and one article was published at the time of the 1987 outbreak (Table 1). While compiling this review, no projects or reports could be found in relation to crayfish or crayfish plague in Northern Ireland.

In Ireland, the first crayfish and crayfish plague survey was completed in 2016, assessing the Erne catchment around the initial outbreak site in 2015. This survey, conducted by the Marine and Freshwater Research Centre at Atlantic Technological University (ATU), Galway (formally Galway-Mayo Institute of Technology), was the first to implement an eDNA methodology for *Ap. astaci* over August and September (2016) in Ireland [18]. Since then, eDNA has become the standard method to detect the pathogen in Ireland [20,21]. The following year, in 2017, ATU undertook a comprehensive national crayfish survey [19]. This survey aimed to determine the distribution of crayfish in 17 special areas of conservation across Ireland. It employed a combination of traditional methods, including hand searching, sweep netting, and overnight baited trapping at 123 sites. The focus was on *A. pallipes*, NICS, and potential crayfish mass mortality events. Notably, environmental DNA techniques were not used during the 2017 survey.

Although no mass mortality events were discovered among the 17 SACs during the 2017 survey, at the same time, several reports of crayfish mass mortalities were reported to the NPWS. These mass mortality events were confirmed to have been caused by *Ap. astaci* in each case. In response, the National Crayfish Plague Surveillance Program (NCPSP) was launched for 2018–2019 crayfish plague monitoring. During this survey, eDNA alone was utilised to track the spread and prevalence of *Ap. astaci* nationwide. During the 2018/2019 NCPSP surveys, 608 water samples were taken from across 28 catchments [20]. Post-filtration, eDNA was extracted and analysed for *Ap. astaci* using qPCR testing (following Vrålstad et al., 2009) [22]. Dead crayfish collected during the survey were also tested with the same qPCR protocol. Out of the twenty-eight catchments, eight tested positive for *Ap. astaci* from DNA extracted from dead crayfish or extracted eDNA (see Supplementary Materials S1): Suir, Barrow, Shannon 26G, Corrib, Shannon Estuary South, and three other Shannon catchments (26A, 26B, and 26D).

The NCPSP program was extended into 2020/2021, during which 738 water samples were collected. Specifically, 168 sites were sampled once, and 30 sites were revisited for repeat sampling. The pathogen was detected in 29 sites, 23 being new site detections. Notably, the 23 new positive sites were identified within nine catchments, including three new catchments (Moy, Sligo, and Shannon 26C) that were previously free of crayfish plague. Interestingly, the Shannon 25A catchment tested negative for *Ap. astaci* using eDNA, but four dead crayfish from the River Clodiagh within the catchment tested positive for the crayfish plague pathogen. Cumulatively, these data show a 59% increase in *Ap. astaci* positive sites between the 2018/2019 and 2020/2021 survey periods. In 2022, the program was extended for a further four years, but no recent data (post-2021) have been released as of December 2023. Data for the sites where *Ap. astaci* was positively detected, including site details, are presented in Supplementary Materials S2.

### 2.3. Reservoir Species and Alternative Host of Aphanomyces astaci

*Aphanomyces astaci* is primarily known as a pathogen of freshwater crayfish but has been observed to infect other freshwater decapods. Some freshwater-inhabiting crabs such as the Chinese mitten crab (*Eriocheir sinensis*) are known carriers of *Ap. astaci*, likely obtained from coexisting crayfish populations, and can transmit the pathogen to susceptible crayfish species [23,24,25,26]. Although *E. sinensis* was reported in Ireland in 2006, its limited sightings and proximity in Waterford harbour suggest a minimal role in *Ap. astaci’s* current distribution in Ireland [27]. Therefore, Chinese mitten crabs are unlikely to be involved in the current distribution of *Ap. astaci* in Ireland.

Ornamental freshwater shrimps like *Macrobrachium dayanum* and *Neocaridina davidi*, established in thermally polluted German streams [28], show resistance to *Ap. astaci* and may facilitate its transmission [24]. However, those freshwater shrimp species have not been recorded in the wild in Ireland. Studies in the UK on *Gammarus pulex* (Malacostraca), a freshwater shrimp long introduced to Ireland, indicate no susceptibility to *Ap. astaci* [29]. However, transmission studies were not completed and the potential of native freshwater species in Ireland to tolerate non-symptomatic and chronic infections certainly warrants further research utilising modern molecular genetic techniques.

The presence of invasive freshwater decapods such as *E. sinensis* in the south of Ireland, and their potential to transmit *Ap. astaci*, underscores the need for research to determine if common freshwater organisms in Ireland can also act as vectors for the pathogen.

### 2.4. The Genetic Lineages of Aphanomyces astaci in Ireland

The attempts to determine the genetic lineages of *Ap. astaci* in Ireland (Supplementary Materials S1) are provided here with the caveat that those data have not been assessed by peer review and the raw data to assess the validity of those assays have not yet been provided by the NCPSP.

Determining the genetic lineages of the pathogen at sites of infection can provide insights into the introduction and spread of *Ap. astaci* and could inform future conservation measures. Five genotypes of *Ap. astaci* were previously defined (A-E) from pure isolates using the random amplification of polymorphic DNA-PCR [30]. A PCR assay was later designed to determine the same genotypes following the whole genome sequencing of the isolates [31]. A more sensitive qPCR assay was later designed to identify the same genotypes [32] and was utilised by the NCPSP in the 2020–2021 NCPSP report. At the same time, genotyping was also conducted using microsatellites [33] and mitochondrial DNA Sanger sequencing [34]. As stated before, the data and results for these analyses have not yet been peer reviewed. Notwithstanding, utilising several published assays, the results consistently showed that multiple genetic lineages have been recorded in Ireland. This is particularly interesting because the C genotype was reported from the same tissue sample in both NCPSP reports (Figure S1; Table S2). The C genotype has never been reported in Europe and so this result requires substantial evidence.

The majority of genetic sampling was completed using tissue samples. Genotyping from eDNA using qPCR alone is not yet a standard method due to variable DNA concentrations at sites and is not routinely used. However, genotyping would be possible where the *Ap. astaci* DNA concentration is sufficient in the environment. Those analyses require review, but the majority of genotyping attempts were performed on tissue samples (Table S2) and can be considered reliable and preliminary until the NCPSP publishes their results in a peer-reviewed academic journal. The development of modern and sensitive assays including that reported by Di Domenico (2021) could become standard in the future.

Considering that three assays were performed, and a number of genotypes were identified, it can be considered highly likely that *Ap. astaci* has been introduced into Ireland independently on a number of occasions.

For this review, personal communication with the Northern Irish authority stated that the lineages of *Ap. astaci* could not be determined because of the storage conditions of the samples.

Given the varying results presented in the two NCPSP reports, the lack of peer review and accountability for those data, and the lack of data from NI, genotyping of all samples should be outsourced to expert labs. This would validate the NCPSP’s results but also help ascertain the genetic lineages in Northern Ireland.

### 2.5. Limitations of the Surveys Conducted

The reports published on crayfish plague in Ireland lack standardisation and are difficult to compare directly regarding sampling effort. However, the latter two NCPSP reports are more aligned than the initial surveys. The survey conducted by ATU in 2016 was the most thorough, albeit the smallest, covering an area centred around the initial outbreak site and limited to the Erne catchment. The 2016 survey included both traditional hand searching, sweep netting, baited overnight trapping, and eDNA analysis. The 2017 survey was aimed at determining crayfish abundance and distribution in 17 special areas of conservation in Ireland. The 2017 report included results for overnight trapping, hand searching, and sweep netting; the search of NICS and mass mortality events were included, but eDNA samples were not analysed. The two NCPSP survey periods were borne from the experience of the 2016 survey. The primary limitation of the NCPSP is the lack of conventional surveys and reliance on eDNA to detect *Ap. astaci* and *A. pallipes*. The 2018–2019 NCPSP surveys only used eDNA testing, while the 2020–2021 NCPSP performed traditional surveys at four sites over two years. However, the NCPSP did sample an impressive number of sites across Ireland.

## 3. The Distribution of *Aphanomyces astaci* across Ireland

Between 2015 and 2023, 18 water catchments tested positive for *Ap. astaci* in Ireland. However, few details have been made public since the 2021 report was published (in 2022). Since then, additional *Ap. astaci* outbreaks have been confirmed in Ireland and Northern Ireland; thus, we can only mention these events briefly.

Where data are available, we describe a comprehensive overview of the timeline of detection, the distribution, and crayfish status within these catchments. We begin with the Erne catchment as it was the first recent site of mass mortality attributed to *Ap. astaci.* We then alphabetically list the subsequent catchments that tested positive for *Ap. astaci* at least once between 2015 and 2021. Details for water catchments were found on the website catchments.ie, provided by the Irish government. Catchments are provided with their individual identifying IDs, e.g., Erne (ID 36). Large catchments such as the Shannon are divided into subcatchments with identifying letters in their codes, e.g., Lower Shannon 25A subcatchment and Lower Shannon 25C subcatchment.

### 3.1. The Erne Catchment

**Figure 3:**
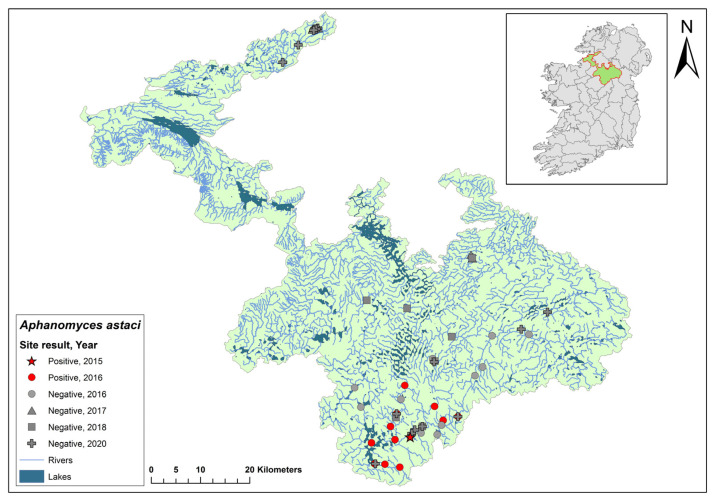
Sites surveyed for *Aphanomyces astaci* in the Erne catchment. Red star indicates the initial site of the Bruskey River crayfish plague event in Co. Cavan. Symbols indicate sampling years; red symbols indicate sites positive for *Ap. astaci*; grey symbols indicate sites negative for *Ap. astaci*.

The Erne catchment (ID 36) spans an area of 4415 km^2^ across Ireland (2512 km^2^) and Northern Ireland (1903 km^2^), and fresh water from the catchment enters the sea in Co. Donegal (Figure 3). The catchment contains 129 rivers, 130 lakes, and 66 groundwater bodies. Following the initial crayfish plague outbreak in July 2015, the NPWS commissioned a study of the Erne catchment to evaluate local crayfish populations and to gauge the persistence of *Ap. astaci* in the area. One year later, the investigation was undertaken by a research team from Atlantic Technological University in August 2016. The team employed hand searching, netting, or overnight trapping to determine the presence of *A. pallipes* and NICS, if possible, throughout the Erne catchment (Figure 3).

An eDNA approach reported by Vrålstad et al. (2009) was adopted to determine the presence of *Ap. astaci* in the catchment [22]. Each of the 24 sites were surveyed twice during the survey. On each visit, the team carried out five-minute visual inspections, hand net searches, and monitored overnight baited traps. Water samples (*n* = 163, +5 negative controls) from all 24 sites were filtered, after which a qPCR analysis was performed to detect *Ap. astaci*. The limit of detection for determining *Ap. astaci* in water samples was determined as Ct 35 (strict positive result) or Ct 40 (relaxed positive result) [22,35].

Of the 24 sites tested by eDNA in 2016, 11 were positive for *Ap. astaci*, with Ct = 35–38. Five of these positive sites contained live, seemingly healthy *A. pallipes* crayfish. The findings from the survey show *Ap. astaci’s* persistence in the catchment both upstream and downstream of the Bruskey River site a year after the first reported outbreak.

As part of the 2018 NCPSP survey, six sites upstream of the 2016 Bruskey river site were assessed using an eDNA analysis for the presence of *Ap. astaci*. All sites were free of the pathogen. A downstream site of the 2015 mass mortality event at Woteraughy Mill and Carrickacleevan remained free of *Ap. astaci* and positive for crayfish. During the 2020 survey, 15 sites were tested around the Erne catchment, including sites around the original mass mortality event recorded in the Bruskey River. All sites tested negative for *Ap. astaci*.

### 3.2. Barrow Catchment

**Figure 4:**
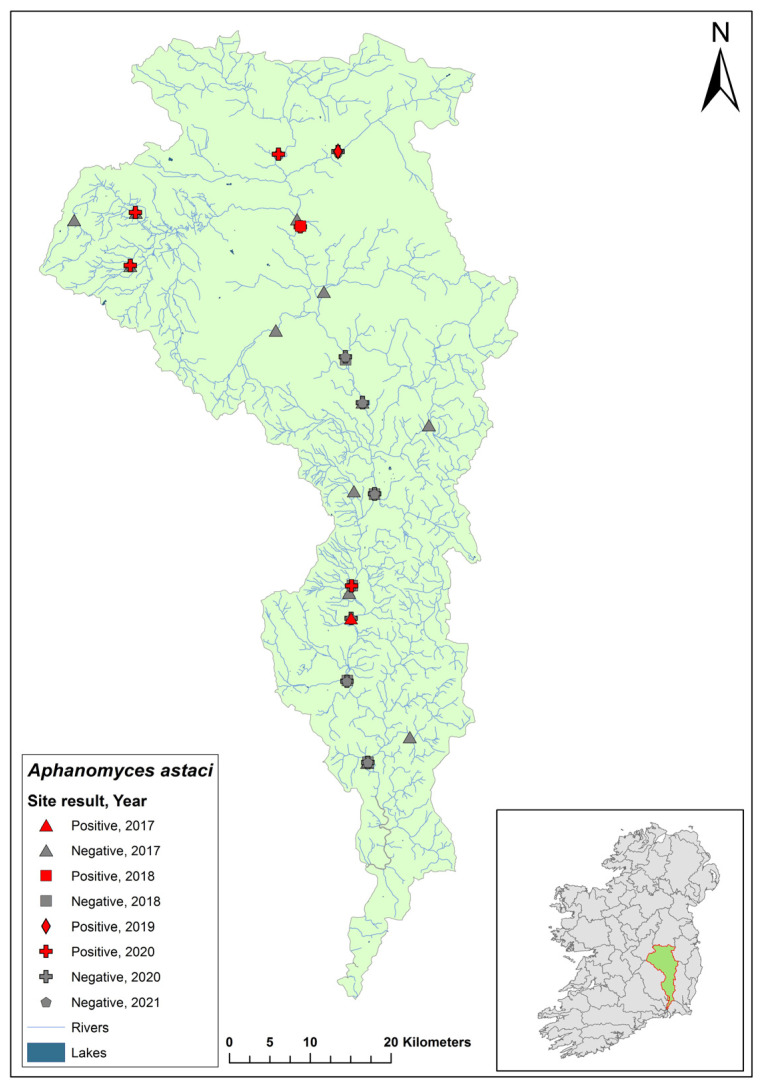
Sites surveyed for *Aphanomyces astaci* in the Barrow catchment. Symbols indicate sampling years; red symbols indicate sites positive for *Ap. astaci*; grey symbols indicate sites negative for *Ap. astaci*.

The Barrow catchment (ID 14) spans an area of 3025 km^2^ containing 149 rivers and six groundwater bodies (Figure 4). In the 2017 crayfish survey, no evidence of NICS or crayfish plague was observed at 12 sites on the River Barrow in Co. Carlow. However, in the same year, crayfish mortalities were reported on the river from Royal Oak Bridge. Dead crayfish samples were assessed and all tested positive for *Ap. astaci*. During the 2018/2019 NCPSP survey, six sites were tested using qPCR testing, and one site at Monasterevin Bridge tested positive for *Ap. astaci* (Ct = 28–33). In 2019, a mortality event was reported in the catchment on the River Slate in Co. Kildare. Tissue samples from crayfish carcases tested positive for *Ap. astaci*. In 2020, twenty-four sites were tested in the catchment and six sites were positive for *Ap. astaci* (Ct = 31–37) and four were positive for *A. pallipes* (Ct = 34–38). In 2021, all 24 sites tested negative for *Ap. astaci* and *A. pallipes*.

### 3.3. Corrib Catchment

**Figure 5:**
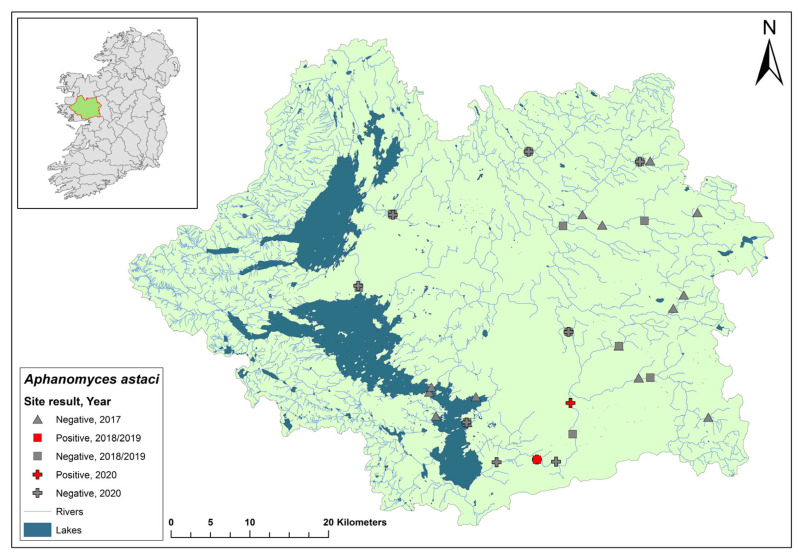
Sites surveyed for *Aphanomyces astaci* in the Corrib catchment. Symbols indicate sampling years; red symbols indicate sites positive for *Ap. astaci*; grey symbols indicate sites negative for *Ap. astaci*.

The Corrib catchment (ID 30) spans an area of 3112 km^2^, comprising 97 rivers, 31 lakes, and 21 groundwater bodies in the west of Ireland (Figure 5). No evidence of crayfish plague was observed at 14 sites in the 2017 survey. The NCPSP sampled six sites in the Corrib catchment in 2018 and twelve sites in 2019. The report states that one site, in Claregalway on the River Clare, Co. Galway, tested positive for *Ap. astaci* in 2018 (Ct = 35) and again in 2019 (Ct = 32–36). In 2020, a site at Corrofin, Co. Clare, tested positive for *Ap. astaci* (Ct = 37). Additionally, *A. pallipes* DNA was detected in the same samples (Ct = 35). Standard ecological sampling was not conducted to confirm the presence of *Ap. astaci* in the catchment and this site was not tested in 2021.

### 3.4. Moy & Killala Bay Catchment

**Figure 6:**
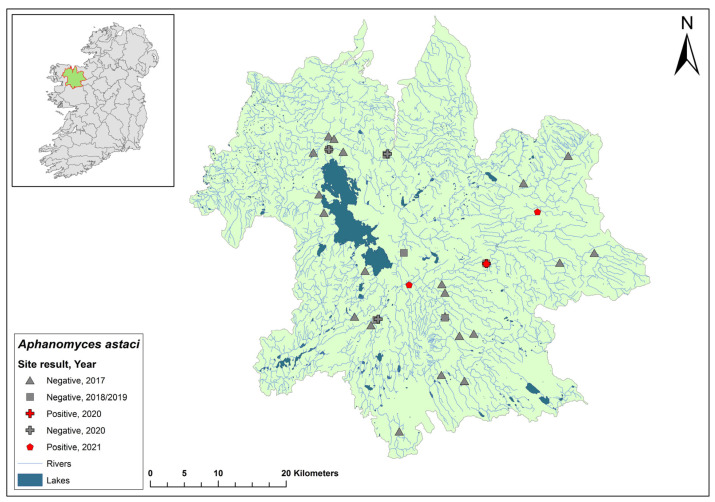
Sites surveyed for *Aphanomyces astaci* in the Moy & Killala Bay catchment. Symbols indicate sampling years; red symbols indicate sites positive for *Ap. astaci*; grey symbols indicate sites negative for *Ap. astaci*.

The Moy & Killala Bay catchment (ID 34) spans 2345 km^2^ containing 115 rivers, 19 lakes, and 37 groundwater bodies in the west of Ireland (Figure 6). A total of 21 sites on the River Moy were assessed in the 2017 survey and no evidence of crayfish plague was observed. Six sites assessed during the first NCPSP survey period also tested negative. However, when assessed in 2020, one site of four at Cloonacannana tested positive for *Ap. astaci* (Ct = 36). In 2021, Cloonacannana and two other sites, one up- and one downstream, tested positive (Ct = 35–36) but no other sites were tested. From the same *Ap. astaci* positive sample sites in 2020 and 2021, positive detections of *A. pallipes* were also made via qPCR. The presence of both *A. pallipes* and *Ap. astaci* over two years at one site could indicate a low virulence strain of *Ap. astaci* at this site.

### 3.5. Nore Catchment

**Figure 7:**
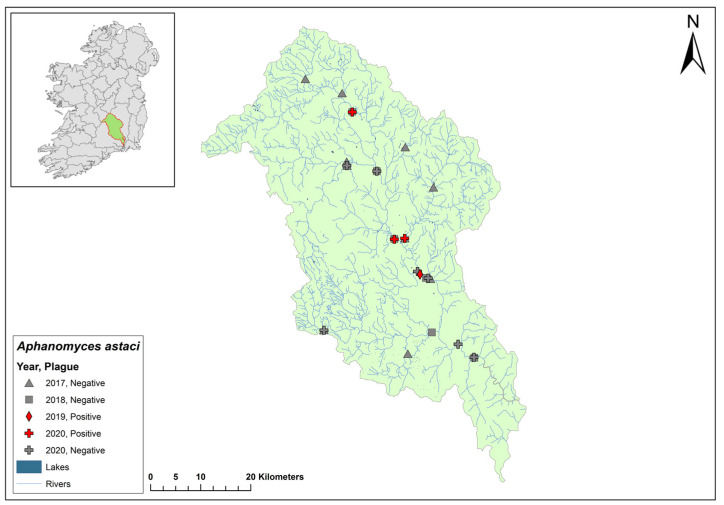
Sites surveyed for *Aphanomyces astaci* in the Nore catchment. Symbols indicate sampling years; red symbols indicate sites positive for *Ap. astaci*; grey symbols indicate sites negative for *Ap. astaci*.

The Nore catchment (ID 15) spans an area of 2595 km^2^ in southeast Ireland and contains 123 rivers, no lakes, and 48 groundwater bodies (Figure 7). The Nore catchment joins the River Barrow before entering the sea. Ten sites on the Nore showed no evidence of crayfish plague during the 2017 survey. Environmental DNA samples from the Nore catchment in 2018 tested negative for *Ap. astaci*. However, in 2019, dead crayfish were sampled from Canal Walk on the River Nore in Kilkenny city, and all tested positive for *Ap. astaci.* In 2020, eDNA showed three of eleven sites tested were confirmed positive for *Ap. astaci* (Ct = 37–38) and *A. pallipes* (Ct = 36–36) at the same time. The Nore catchment was not sampled in 2021.

### 3.6. Shannon Estuary South Catchment

**Figure 8:**
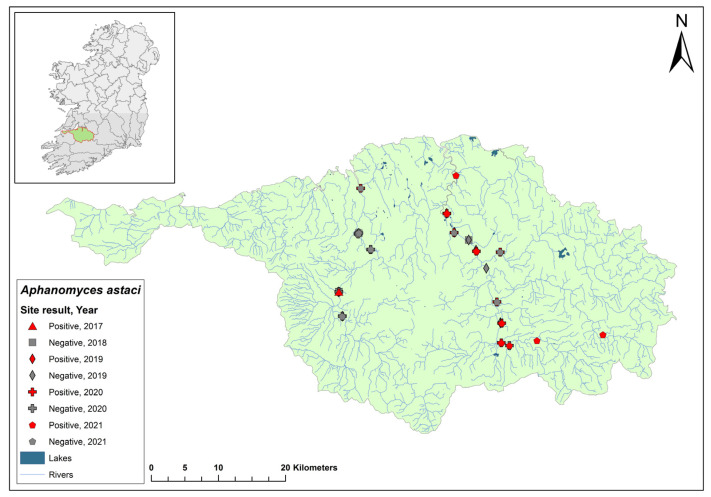
Sites surveyed for *Aphanomyces astaci* in the Shannon Estuary South catchment. Symbols indicate sampling years; red symbols indicate sites positive for *Ap. astaci*; grey symbols indicate sites negative for *Ap. astaci*.

The Shannon Estuary South catchment (ID 24) spans an area of 2033 km^2^ south of the River Shannon estuary and contains 95 rivers, two lakes, and 46 groundwater bodies in the west of Ireland (Figure 8). Dead crayfish were sampled from the River Deel in the catchment following reports of crayfish mortalities in 2017. All samples tested positive for *Ap. astaci*. In 2018, the same sites with additional locations were resampled and all tested negative for *Ap. astaci*.

In 2019, a crayfish mass mortality event was reported at Castleroberts Bridge on the River Maigue within the catchment (Ct = 37). Two sites, one near the mass mortality event and one upstream, tested negative for the crayfish plague pathogen. Additional sites on the rivers tested positive (Ct = 27–30); the same samples also tested positive for *A. pallipes* (Ct = 32–34).

In 2020, 13 sites in the catchment tested positive for both *Ap. astaci* (Ct = 27–40) and *A. pallipes* (Ct = 27–40). In 2021, six sites tested positive for both *Ap. astaci* (Ct = 34–39) and *A. pallipes* (Ct = 32–37). Of these, four sites consistently tested positive for both over the 2020 and 2021 sampling period. A site at Askeaton Main Street was tested in June and November of 2020 and 2021. Both sampling timepoints in 2020 tested positive (June – Ct = 36, November – Ct = 37), while the same time points tested negative in 2021. From the same samples, *A. pallipes* was identified at the site in June (Ct = 40) and November (Ct = 38) in 2020 but not 2021. These data imply that *Ap. astaci* devastated the *A. pallipes* population at the site between November 2020 and June 2021.

### 3.7. Lower Shannon (Brosna) 25A Catchment

**Figure 9:**
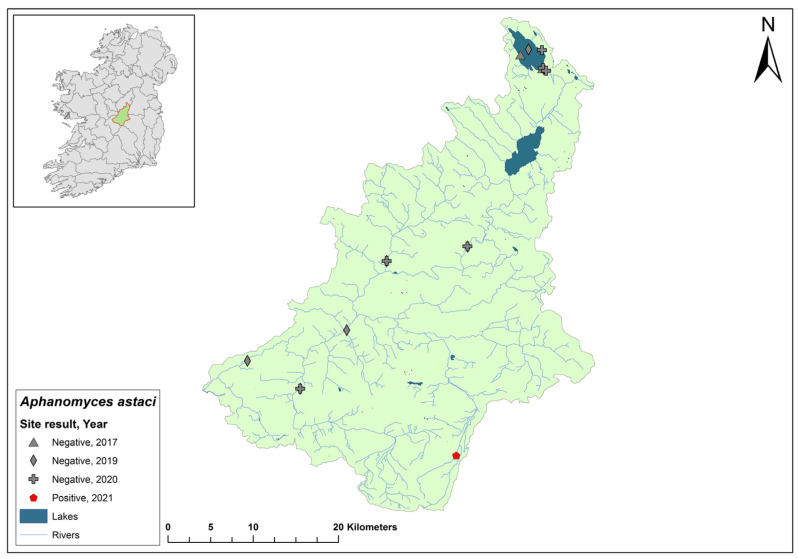
Sites surveyed for *Aphanomyces astaci* in the Lower Shannon 25A catchment. Symbols indicate sampling years; red symbols indicate sites positive for *Ap. astaci*; grey symbols indicate sites negative for *Ap. astaci*.

The Lower Shannon catchment (ID 25A) spans an area of 1248 km^2^ containing 62 rivers, four lakes, and 32 groundwater bodies (Figure 9). In 1987, *Ap. astaci* was diagnosed as the cause of a mass mortality event at Lough Owel, but no samples remain. No further spread of the pathogen was detected. In 2017, Lough Owel was sampled without any indications of crayfish plague at two sites and had some of the healthiest populations of crayfish across Ireland. In the 2018/2019 NCPSP surveys, seven sites tested negative in the Shannon 25A catchment. Again, in 2020, six sites tested negative. However, in 2021, four dead crayfish were reported in the catchment at Clonaslee on the River Clodiagh, Co. Laois. All four crayfish tested positive for *Ap. astaci*. No further detection of *Ap. astaci* has been reported in the catchment. Crayfish were detected in the catchment during the 2018/2019 (Ct = 36–39) and 2020/2021 (Ct = 35–38) surveys.

### 3.8. Lower Shannon (Lough Derg) 25C Catchment

**Figure 10:**
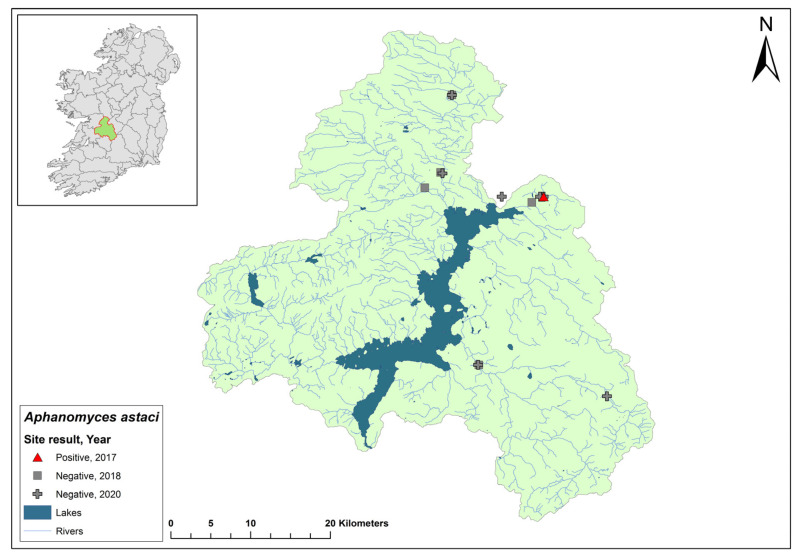
Sites surveyed for *Aphanomyces astaci* in the Lower Shannon 25C catchment. Symbols indicate sampling years; red symbols indicate sites positive for *Ap. astaci*; grey symbols indicate sites negative for *Ap. astaci*.

The Lower Shannon catchment (ID 25C) spans an area of 1820 km^2^ containing 79 rivers, five lakes, and 10 groundwater bodies (Figure 10). In 2017, a research team studying crayfish population genetics from the Marine and Freshwater Research Centre at Atlantic Technological University discovered a crayfish mass mortality event on the River Lorrha in Lorrha village, Co. Tipperary. Dead crayfish samples were taken to the Marine and Freshwater Research Centre, where they tested positive for *Ap. astaci*. The NCPSP assessed six sites in the Shannon 25C catchment in 2018, including Lorrha village, and in 2020, seven sites were assessed in the catchment. All sites following the 2017 mass mortality event were free of *Ap. astaci*. Using eDNA, *A. pallipes* were detected (Ct = 37–38) in Lorrha village in the 2018/2019 report, and again in 2020 (Ct = 35–37). The catchment was not tested in 2021.

### 3.9. Ulster Blackwater Catchment

**Figure 11:**
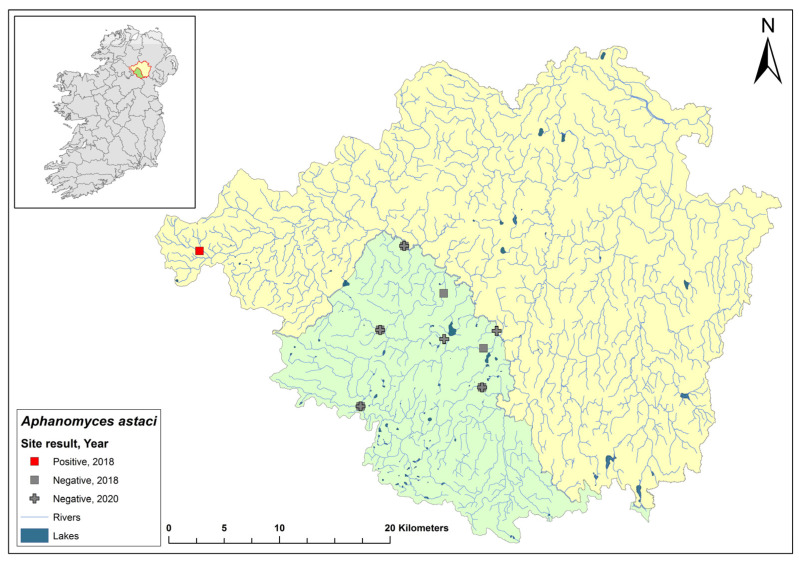
Sites surveyed for *Aphanomyces astaci* in the Ulster Blackwater catchment. Green indicates the area within Ireland; yellow indicates area within Northern Ireland; symbols indicate sampling years; red symbols indicate sites positive for *Ap. astaci*; grey symbols indicate sites negative for *Ap. astaci*.

The Ulster Blackwater is a cross-border catchment spanning a total area of 1491 km^2^, 1097 km^2^ in Northern Ireland, and 393.8 km^2^ in Ireland (Figure 11). In September 2018, dead crayfish were found during a routine field survey at the headwater of the River Blackwater. The specimens tested positive for *Ap. astaci*. Following this discovery, there has been no publicised information regarding further crayfish mortalities in the area, nor have there been updates on any subsequent efforts to evaluate the full impact of crayfish plague in the catchment.

### 3.10. Upper Shannon (Lough Allen) 26A Catchment

**Figure 12:**
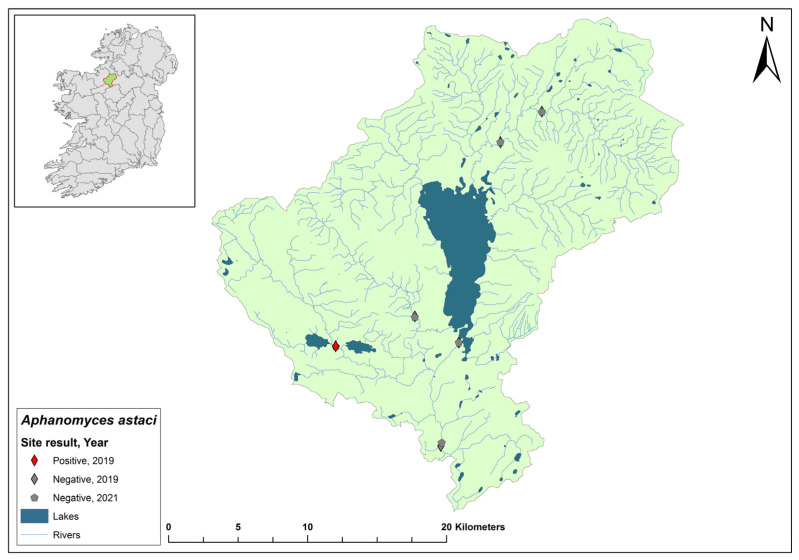
Sites surveyed for *Aphanomyces astaci* in the Upper Shannon 26A catchment. Symbols indicate sampling years; red symbols indicate sites positive for *Ap. astaci*; grey symbols indicate sites negative for *Ap. astaci*.

The Upper Shannon catchment (ID 26A) spans an area of 604 km^2^ containing 25 rivers, eight lakes, and 18 groundwater bodies (Figure 12). Six samples from the Shannon 26A catchment were sampled in 2019. One sample collected at Ballyfarnon on the Feorish River, Co. Roscommon, tested positive for *Ap. astaci* (Ct = 35). At the same time, crayfish were detected using eDNA (Ct = 38–40). During the 2020/2021 surveys, all of six sampled sites in the Shannon 26A catchment were negative for *Ap. astaci* and two sites tested positive for *A. pallipes* using eDNA (Ct = 34–35).

### 3.11. Upper Shannon (Boyle) 26B Catchment

**Figure 13:**
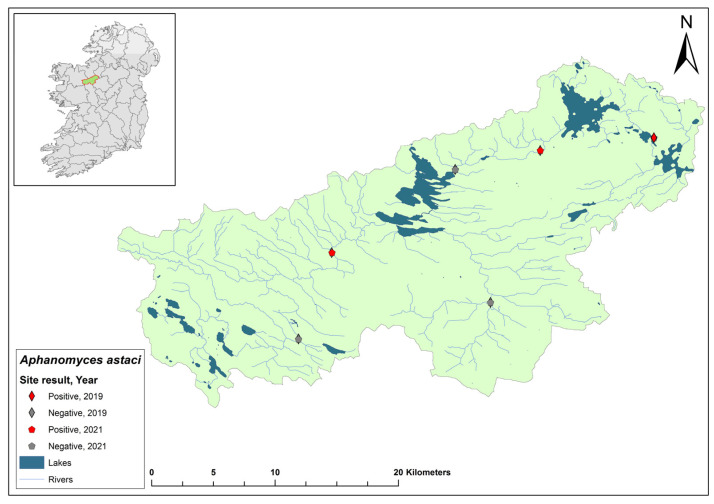
Sites surveyed for *Aphanomyces astaci* in the Upper Shannon 26B catchment. Symbols indicate sampling years; red symbols indicate sites positive for *Ap. astaci*; grey symbols indicate sites negative for *Ap. astaci*.

The Upper Shannon catchment (ID 26B) spans an area of 674 km^2^ containing 28 rivers, 15 lakes, and eight groundwater bodies (Figure 13). Six sites from the Shannon 26B catchment were sampled in 2019. One sample, collected at Cootehall Bridge, Co. Roscommon, tested positive for the pathogen (Ct = 35–36). No sites were tested in 2020. Of the six sites assessed in 2021, four sites remained free of *Ap. astaci*. However, two sites, one at Bridge West (Ct = 35) and another at Boyle Footbridge (Ct = 33), tested positive for *Ap. astaci*. Moreover, *A. pallipes* was also detected at both sites using eDNA (Ct = 37 and 36, respectively).

### 3.12. Upper Shannon 26C

**Figure 14:**
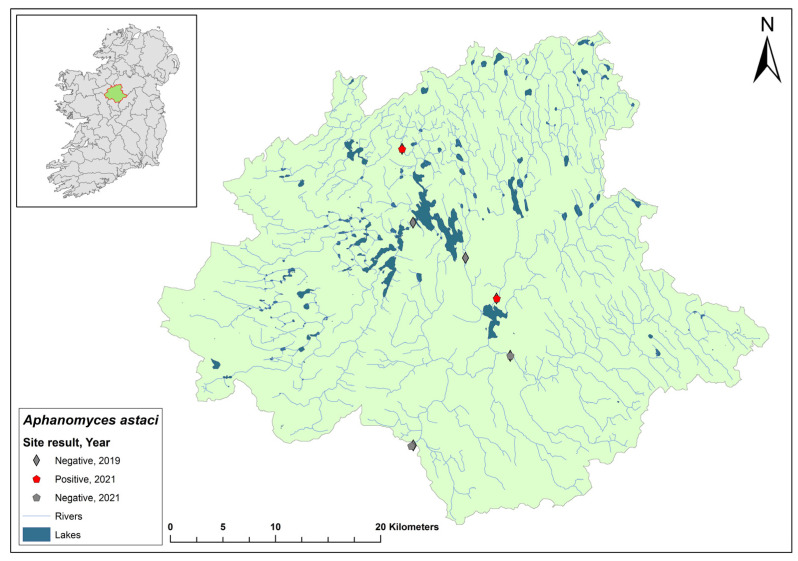
Sites surveyed for *Aphanomyces astaci* in the Upper Shannon 26C catchment. Symbols indicate sampling years; red symbols indicate sites positive for *Ap. astaci*; grey symbols indicate sites negative for *Ap. astaci*.

The Upper Shannon catchment (ID 26C) spans an area of 1500 km^2^ containing 58 rivers, 23 lakes, and 15 groundwater bodies (Figure 14). In the 2019 survey, six sites were assessed in the Shannon 26C catchment and all tested negative for the pathogen. In 2021, two of the same sites, Drumsna (Ct = 37) and Rinn Marina (Ct = 38), tested positive for *Ap. astaci*, while two others tested negative. At the same time, eDNA did not detect *A. pallipes* at any of the sites in the catchment.

### 3.13. Upper Shannon (Suck) 26D Catchment

**Figure 15:**
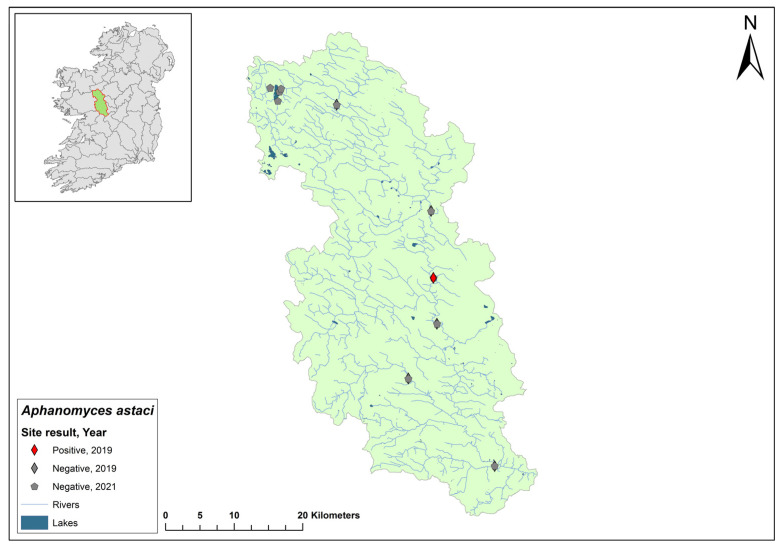
Sites surveyed for *Aphanomyces astaci* in the Upper Shannon 26D catchment. Symbols indicate sampling years; red symbols indicate sites positive for *Ap. astaci*; grey symbols indicate sites negative for *Ap. astaci*.

The Upper Shannon catchment (ID 26D) spans an area of 1598 km^2^ containing 58 rivers, one lake, and 17 groundwater bodies (Figure 15). Six sites were selected for assessment in the Shannon 26D catchment in 2019. One site, Mount Talbot on the River Suck in Co. Roscommon, tested positive for *Ap. astaci* (Ct = 36–38). However, no crayfish mortalities attributed to crayfish plague have been identified within the catchment. Sampling was not conducted in the catchment in 2020. In 2021, 12 sites were assessed and tested negative for the *Ap. astaci*, but six were positive for *A. pallipes* (Ct = 30–37).

### 3.14. Shannon 26G Catchment

**Figure 16:**
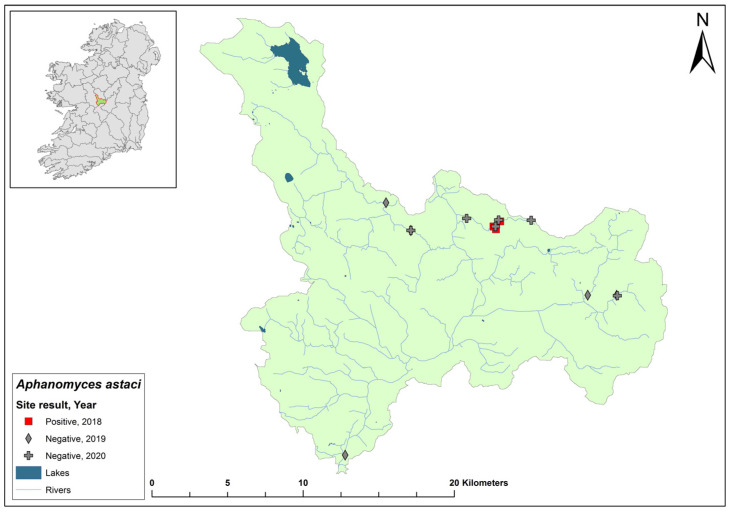
Sites surveyed for *Aphanomyces astaci* in the Upper Shannon 26G catchment. Symbols indicate sampling years; red symbols indicate sites positive for *Ap. astaci*; grey symbols indicate sites negative for *Ap. astaci*.

The Upper Shannon catchment (ID 26G) spans an area of 383 km^2^ and contains 13 rivers, 12 lakes, and one groundwater body (Figure 16). In 2018, reports of crayfish mortalities were reported in the River Al in the Shannon 26G catchment. Three sites were sampled and all tested positive for *Ap. astaci* (Ct = 32–35). One year later, the sampling sites were inaccessible due to flooding. Six other sites in the Shannon 26G catchment were monitored and tested negative for *Ap. astaci*. Seven sites were assessed in Shannon 26G catchment in 2020 and were negative for *Ap. astaci*. At the same time, *A. pallipes* were identified using eDNA at four of the six sites tested (Ct = 37–40). The catchment was not assessed in 2021.

### 3.15. Sligo Bay & Drowse Catchment

**Figure 17:**
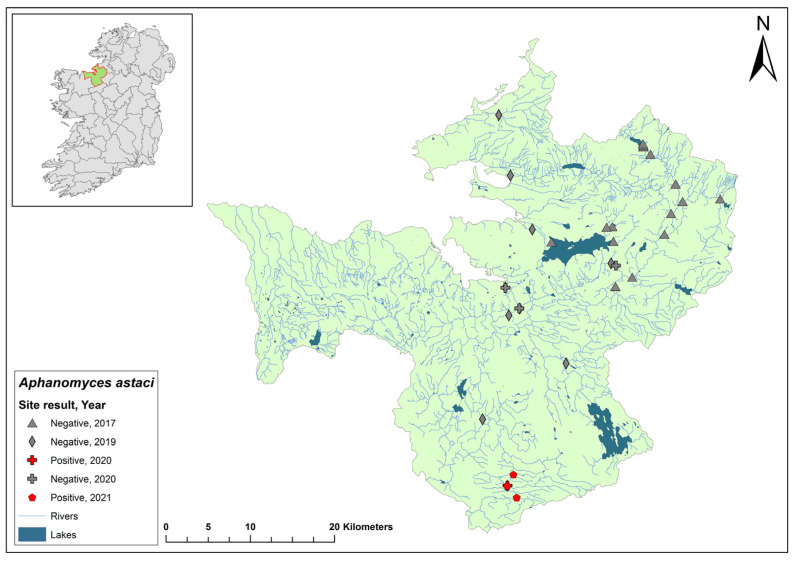
Sites surveyed for *Aphanomyces astaci* in the Sligo Bay & Drowse catchment. Symbols indicate sampling years; red symbols indicate sites positive for *Ap. astaci*; grey symbols indicate sites negative for *Ap. astaci*.

The Sligo Bay & Drowse catchment (ID 35) spans an area of 1866 km^2^ and contains 70 rivers, 18 lakes, and 25 groundwater bodies in the northwest of Ireland (Figure 17). Fifteen sites were monitored in the 2017 survey and no evidence of NICS or crayfish plague were found. Eleven sites were assessed in the 2019 survey and all tested negative for *Ap. astaci*. In 2020, one site of the four sites tested, Gurteen, tested positive for *Ap. astaci* (Ct = 35) as well as for *A. pallipes* (Ct = 33). In 2021, three sites, all around Gurteen, tested positive for the pathogen (Ct = 30–32) and *A. pallipes* (Ct = 32–36), but no other sites were assessed.

### 3.16. Suir Catchment

**Figure 18:**
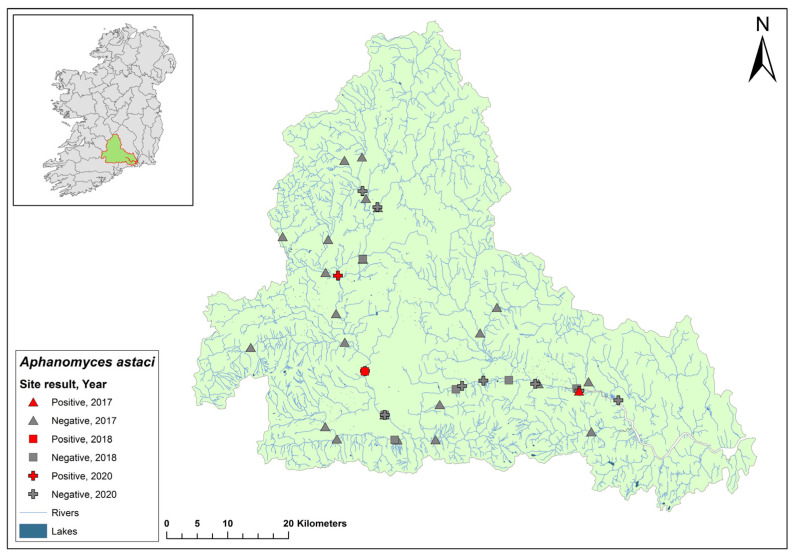
Sites surveyed for *Aphanomyces astaci* in the Suir catchment. Symbols indicate sampling years; red symbols indicate sites positive for *Ap. astaci*; grey symbols indicate sites negative for *Ap. astaci*.

The Suir catchment (ID 16) spans an area of 3542 km^2^ and contains 168 rivers, seven lakes, and 43 groundwater bodies in the south of Ireland (Figure 18). The second recorded outbreak of crayfish plague in Ireland occurred in 2017 on the River Suir, Co. Tipperary. Over a 24 km stretch of the river in the catchment, crayfish losses were estimated to be around 400,000 animals. During the 2017 crayfish survey, 22 sites that were sampled in the Suir catchment all tested negative for crayfish plague. Of the seven sites assessed in the Suir catchment in 2018, Cahir Bridge on the River Suir tested positive for *Ap. astaci* (Ct = 36–36), while Carrick-on-Suir tested negative, having tested positive the previous year. In 2020, one site out of the ten sampled in the Suir catchment, River Multeen (Ct = 36), tested positive for *Ap. astaci*. The catchment was not assessed in 2021.

### 3.17. Outbreaks between 2021 and 2023

**Figure 19:**
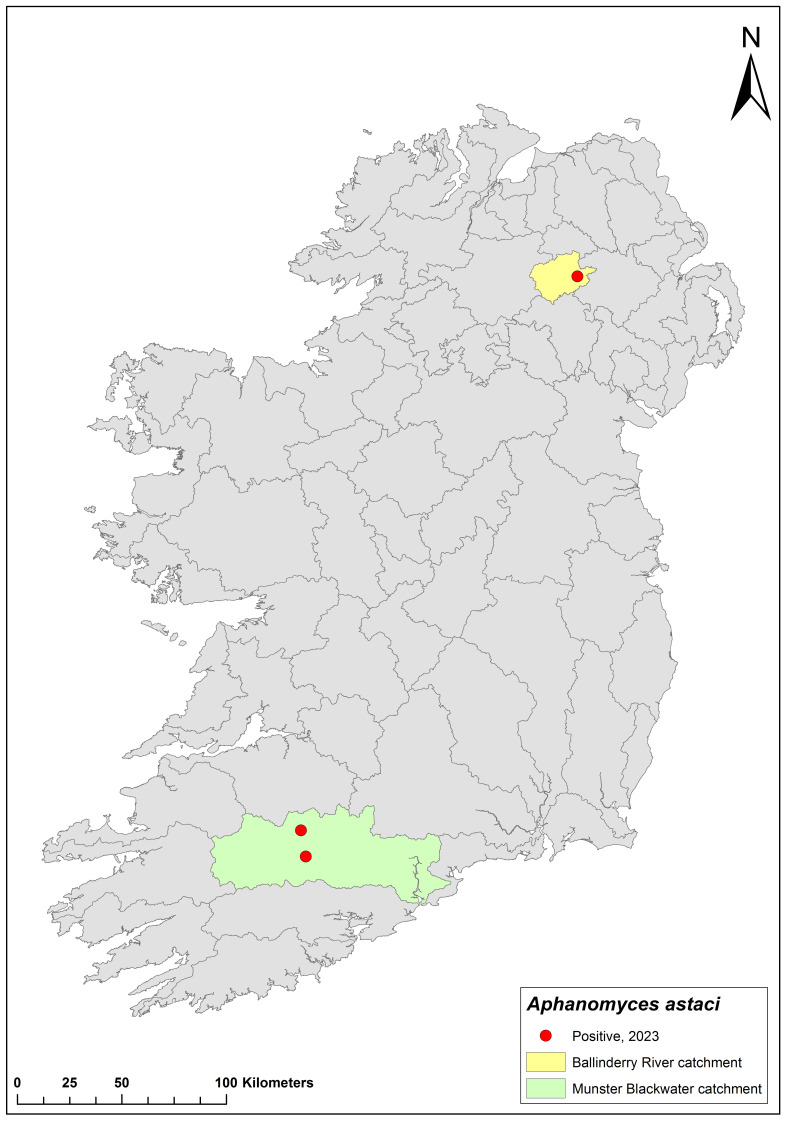
*Aphanomyces astaci* outbreaks recorded in 2023. Yellow area represents the Upper Ballinderry River catchment in Northern Ireland; green area represents the Munster Blackwater catchment in Ireland.

Irish authorities have not released any crayfish plague sampling data since the completion of the NCPSP 2021 survey, and Northern Irish authorities have not released any official data. However, crayfish plague events persist and although none were recorded in 2022, two outbreaks have been confirmed by authorities in Ireland and Northern Ireland in 2023. In July, *Ap. astaci* was detected in the Munster Blackwater in Ireland. In September, an outbreak was reported in the upper Ballinderry River catchment in Northern Ireland (Figure 19). To date, these two events mark the southernmost and northernmost expansions of *Ap. astaci* in Ireland, respectively.

As of 9 October 2023, no record of *Ap. astaci* has been logged with the National Biodiversity Network (NBN) Atlas of the United Kingdom of Great Britain and Northern Ireland. Historical records are available from the late 1960s and the 1970s for several other *Aphanomyces spp*., including *Ap. cochlioides*, *Ap. euteiches*, *Ap. laevis*, *Ap. parasiticus*, *Ap. scaber*, and *Ap. stellatus.* Two records of *Ap. astaci* that were detected using eDNA in England in September 2023 were logged on the iRecord system, which acts as a hub for experts to confirm records before they are logged in the Centre for Environmental Data and Recording (CEDaR) database and are then published in the NBN atlas. However, neither record of *Ap. astaci* from Northern Ireland appears to have been recorded. The National Biodiversity Data Centre in Ireland contains records of ten outbreak events from the July 2015 crayfish plague event on the Bruskey River to the April 2019 event on the River Maigue; no records have been added since April 2019.

## 4. Non-Indigenous Crayfish Species

Globally, there are over 600 described species of freshwater crayfish [36]. On the island of Ireland, all except the naturalised species *A. pallipes* are considered NICS [14]. Some NICS possess traits that make them highly invasive, including rapid growth, large size, and high fecundity, that would enable them to outcompete native *A. pallipes* within its Irish range [2,25]. Many NICS have been imported and released in Europe, several establishing populations and devastating native crayfish populations and habitats. The spiny-cheek crayfish (*Faxonius limosus*), virile crayfish (*F. virilis*), signal crayfish (*Pacifastacus leniusculus*), red swamp crayfish (*Procambarus clarkii*), and marbled crayfish (*Pracambarus virginalis*) are all established in Europe, compete with native crayfish, and can transmit *Ap. astaci* [2,37,38]. Given Ireland’s mild temperate oceanic climate, many NICS would likely thrive in Irish freshwaters.

### 4.1. Legislative Changes Regarding Non-Indigenous Crayfish Species

Ireland implemented legislative changes with the “S.I. No. 354/2018—European Union (Invasive Alien Species) (Freshwater Crayfish) Regulations 2018 (SI 354/18)” that came into effect on 18 September 2018 [39]. The regulation was designed to mitigate the risk of disease transmission from NICS by prohibiting the trade of five species, including *F. limosus*, *F. virilis*, *P. leniusculus*, *P. clarkii*, and *P. virginalis* species that are well established in Europe. The legislation expressly forbids the intentional release of these species into natural habitats and constrains the intentional possession, transportation, sale, breeding, exchange, and ornamental use of live specimens, barring specific exemptions, including research-related activities.

Critical transport stipulations mandate that deceased and live specimens of these crayfish should be managed with precautions to impede potential reproduction or escape. Moreover, any transportation of soil or freshwater from areas known to host these species is regulated, ensuring the containment of their spread. However, the testing of soil and water for *Ap. astaci* using qPCR or other molecular methods is not specified. The legislation cautions against any actions that might inadvertently aid the reproduction, spread, or establishment of these crayfish species, unless explicitly sanctioned. Furthermore, the 2018 legislation underscores adherence to eradication measures: landowners or custodians are obligated to comply with any directives from the Minister (for Culture, Heritage, and the Gaeltacht) geared towards the control or elimination of these species.

A significant aspect of the legislation is its emphasis on cooperation and accurate reporting; failures to assist, obstructions of authorised personnel, or deliberate misinformation are deemed violations. Penalties and fines for breaching this legislation could reach a maximum of €100,000 and/or imprisonment for up to two years.

### 4.2. Wild, Established Non-Indigenous Crayfish Species in Ireland

The first record of established wild NICS in Ireland was reported in 2019 with the presence of a strong population of Common Yabby (*Cherax destructor*) in Ballyhass Lake, a former quarry in Co. Cork [40]. The species typically requires higher temperatures to survive than are present in Ireland year-round, and Ballyhass Lake is fed by a thermal spring [40]. The stock is estimated to have been present at the site for ten years, while no records of *C. destructor* have been reported elsewhere in the local area. In experimental trials, *C. destructor* was shown to be generally susceptible to *Ap. astaci* infection [41], but some survival was observed after infection with the least virulent *Ap. astaci* strain [42]. Therefore, under favourable conditions, *C. destructor* could transmit *Ap. astaci* in Ireland [42]. It has not been reported whether the Ballyhass lake population of *C. destructor* have been tested for *Ap. astaci*.

The actions to be taken by Irish authorities when populations of NICS are discovered are listed in the 2018 legislation (described in Section 4.1 [35]), designed to protect native habitats and species from NICS, and states the following:

*“Part 5, Eradication, Section 10. (1) Where the Minister confirms the appearance in the State or part thereof of an invasive alien species of crayfish—(a) whose presence in the State was previously unknown… he or she shall… (i) without delay, publish a statement on the internet providing details of the confirmation, and (ii) as soon as practicable but within 3 months of confirmation of the appearance, develop and adopt eradication measures and apply or secure the application of those eradication measures.”*

Considering that *C. destructor* is not specifically listed as one of the five species of concern in the legislation, it is unclear whether any action will be taken to eradicate the population.

### 4.3. No Non-Indigenous Crayfish Species Found during the 2016–2021 Surveys

The 2016 Erne catchment survey assessed the presence of NICS via conventional hand searching, netting, or overnight trapping methods and no NICS were found. Likewise, during the 2017 national crayfish survey, no NICS were found using hand searching, netting, or overnight traps. Subsequently, the NCPSP developed a multiplex qPCR assay (one reaction detecting two or three species of NICS each) to test for eight species of NICS, including the following species: signal crayfish (*P. leniusculus*), noble crayfish (*A. astacus*), spiny-cheek crayfish (*F. limosus*), marbled crayfish (*P. virginalis*), red-swamp crayfish (*P. clarkii*), common yabby (*C. destructor*), narrow-clawed crayfish (*A. leptodactylus*), and virile crayfish (*F. virilis*). However, parameters and validation data of this multiplex assay developed by the Marine Institute have not been published [21]. During the NCPSP monitoring programs, no evidence for NICS was found.

### 4.4. The Sale of Non-Indigenous Crayfish Species in Ireland through the Pet Trade

Ornamental crayfish from the pet trade have been confirmed as carriers of *Ap. astaci* previously and represent a threat physically and by their contaminated water being released into the environment [43,44]. In Ireland, NICS have been available for purchase online through the pet trade. *P. virginalis*, *P. clarkii*, *C. quadricarinatus*, and *Cambarellus patzcuarensis* have been reported for sale in 2015 and 2017 [45,46]. Regardless of the 2018 legislative changes, a search of the popular sales website DoneDeal.ie, on 24 September 2023, showed that Dwarf Red Lobster (*C. patzcuarensis*) were available in County Dublin at time of writing (Supplementary Materials S4; on 5 October the advert was online for 43 days). It is not likely that *C. patzcuarensis* could tolerate the temperate Irish climate, and the threat of the species establishing and outcompeting the white-clawed crayfish is low [47]. Albeit at low levels, *C. patzcuarensis* is a recorded vector of *Ap. astaci* [43]. As *C. patzcuarensis* is not listed on the restricted crayfish list (SI 354/18), it is unlikely that any action can be taken to intervene in its sale.

## 5. Management and Mitigation Strategies

### Passive Monitoring

Authorities in both Ireland and Northern Ireland appear to have taken a laissez-faire non-intervention approach to the management of NICS and *Ap. astaci*. Research funding related to *Ap. astaci* in Ireland has primarily focused on monitoring and determining the spread of the plague pathogen across water catchments; a surveillance programme was established without a mitigation or management programme. Yet, surprisingly, proactive restrictions have not been imposed, such as limiting the movement of wet gear and watercrafts between waterbodies during active mass mortality events. While efforts were taken to publicise the initial plague event and subsequent outbreaks, including a detailed press release by Inland Fisheries Ireland [17], the emphasis has largely been on passive voluntary preventative measures. “Voluntary bans’’ were placed, extended, and lifted on several waterbodies.

The data indicate that the voluntary bans were ineffective at curbing the spread of *Ap. astaci*, and this measure was criticised by stakeholders, including the angling community, for not adequately protecting *A. pallipes* [48,49]. Stakeholders received advice on the “Check, Clean, Dry” protocol when transitioning between watercourses, and similar literature and videos were disseminated online [50,51]. Signs informing the public about crayfish plague and detailing the protocol were prominently placed at high-traffic watercourses. Yet, the continued spread of *Ap. astaci* to new catchments suggests these passive measures have been ineffective. Considering the initial four reported crayfish plague cases in Ireland (spanning from Bruskey River [2015] to River Deel [2017]), each likely presented unique genotypes (Supplementary Materials Table S2), which indicates they were independent introductions. This raises the possibility that the initial outreach campaign did not sufficiently target the international community, particularly tourists engaged in angling and water-based recreational activities in Ireland. This oversight may have inadvertently facilitated the pathogen’s rapid succession and multiple introductions.

The legislative change made in 2018 to prohibit the trade of five NICS in the country is arguably the strongest effort made to protect the freshwater environment but is also lacking. Only five species of NICS were prohibited. Considering Ireland only has a single protected species of freshwater crayfish, the legislation could have been extended to all freshwater crayfish species, as interfering with *A. pallipes* was already prohibited. Neither the established population of *C. destructor* nor *C. patzcuarensis*, recently being sold online, are listed in the 2018 legislation.

Regarding Northern Ireland, similar efforts to those by southern authorities were made online to advertise crayfish plague, with the same “Check, Clean, Dry” protocols [51]. However, there is little evidence of any effort to monitor the distribution of *Ap. astaci* in Northern Ireland and no scientific or grey literature can be found.

## 6. Perspectives and Considerations for the Future

### 6.1. Crayfish Plague Surveys Have Not Informed Crayfish Conservation

The rapid spread of crayfish plague in Ireland, impacting numerous water catchments, highlights a critical gap in conservation efforts across Ireland. In the eight years since the 2015 detection of crayfish plague, 18 water catchments have been affected. Neither Irish nor Northern Irish authorities have implemented timely or active measures that stopped the spread of the disease. The National Crayfish Plague Surveillance Program in Ireland is, as the name states, a surveillance programme to monitor *Ap. astaci’s* distribution. It is important to restate that no effective measures have been implemented to halt crayfish plague’s progression. Regarding Northern Ireland, no program, survey, or report could be found on the topic.

Given that the crayfish plague pathogen can eliminate *A. pallipes* from a site within a few weeks and considering that outbreaks are typically only reported after a mass mortality event, it is likely that by the time the disease is detected, it has already spread throughout an entire site. Therefore, at the stage of detection following a mortality event, there are no viable mitigation measures or restorative solutions available. However, three immediate actions that could reduce the further spread of *Ap. astaci* are (1) an immediate prohibition on the public entering the catchment at or near the mortality event site; (2) where practical, the removal of dead and moribund animals from the site to reduce the number of *Ap. astaci* spores released from infected carcasses [52]); and (3) the implementation of barriers to reduce upstream spread where possible [53,54]. Although these measures would require careful planning and readiness, the occurrence of *Ap. astaci* already calls for an urgent need for coordinated and rapid actions to prevent further mass mortality events.

### 6.2. Effective Management Strategies for Crayfish Conservation

As previously discussed, proactive management might involve legislative changes to restrict public access to areas during mass mortality events. Such measures would enable the swift semi-isolation of crayfish plague sites, facilitated by field officers setting up clear signage at key access points. Moreover, the removal of deceased and dying crayfish could significantly reduce the release of spores into the water, albeit demanding substantial resources. As an island, Ireland has a unique opportunity to return to its crayfish plague-free and NICS-free status. Thus, it is crucial to implement legislation that prohibits the introduction of all live and unprocessed NICS, extending beyond the species already present in Europe. Exceptions should be made only for essential purposes, including scientific research. In the event of a crayfish plague outbreak caused by *Ap. astaci*, the pathogen is likely to die out once all host crayfish perish. After such an outbreak, it would be prudent to undertake a thorough eDNA survey across affected water bodies to determine whether *Ap. astaci* spores remain. Should repeated water tests consistently return negative results, it could then become viable to consider reintroducing *A. pallipes* into these environments. No infection experiments have been performed on Irish populations of *A. pallipes*. However, evidence from Spain showed that different populations of *A. pallipes* from the north of the country had varying levels of survival following infection with *Ap. astaci* [55]. The chronic infection of *Austropotamobius torrentium* spanning several years has been reported from Slovenia [56], while populations of *A. pallipes* from Spain showed variable resistance lasting at least four months in some cases following infection with *Ap. astaci* [55]. If a similar case was to arise among Irish populations of *A. pallipes*, then chronically infected individuals might inhibit the reintroduction of the species. Therefore, where environmental samples indicate the long-term persistence of *Ap. astaci* and *A. pallipes*, the survival and actual infection status of *A. pallipes* should be determined from live samples at those sites over an extended period. 

### 6.3. Conventional Crayfish Sampling

Environmental DNA provides a powerful means to sample a large number of sites for specific species. Although eDNA is a relatively new sampling methodology with extremely powerful detection potential, it possesses inherent weaknesses. These limitations include imperfect sampling, which can occur at any stage of the protocol, contamination, false positive results, false negative results, and biases in sampling and processing (including user bias) [57]. In a field study testing for coldwater crayfish (*Faxonius eupunctus*), positive eDNA detections were shown to increase as one moves further downstream from a population, regardless of abundance [58]. Another study showed that *P. clarkii* crayfish carcasses do not produce levels that are detectable using eDNA [59]. These studies indicate that detecting crayfish and their abundances using eDNA alone is challenging and error prone.

During the NCPSP surveys, certain sites provided surprising results. The Upper Shannon 26D catchment is an example where positive *Ap. astaci* but negative *A. pallipes* results were obtained using eDNA. Gurteen, in the Sligo Bay & Drowse catchment, tested positive for *Ap. astaci* and *A. pallipes* in 2020, and again in 2021. These are two interesting examples that warrant further investigation. Given the NCPSP’s reliance on eDNA to detect *A. pallipes*, contradictory findings, such as the detection of *Ap. astaci* without its host, might suggest false positives. Indeed, false negatives could also be given for the presence of *A. pallipes* and NICS, which are included in the assays in Ireland. Chinese mitten crabs were removed from the assay as it used non-species-specific primers [21]. In such scenarios, traditional survey methods, including hand searching, sweep netting, and overnight bait trapping, should be employed to verify the presence or absence of these species. These methods were notably underutilised during the 2018–2021 surveys. Environmental DNA analyses should complement, rather than replace conventional crayfish sampling, and a predetermined number of sites should be designated for traditional sampling to serve as controls, at a minimum.

It should be noted that in the 2020/2021 NCPSP report, the large number of false positive Chinese mitten crab results could be due to a non-species-specific assay utilised to amplify DNA from amphipods and rotifers, commonly found taxa in European freshwater environments. The report states that a species-specific assay will be employed in future work.

### 6.4. Determining Genetic Lineages of Aphanomyces astaci in Ireland

As mentioned in Section 2.3, considerable efforts appear to have been made to determine the genetic lineages of *Ap. astaci* in Ireland during the NCPSP surveys. Interesting qualitative genetic data have been provided by Irish authorities regarding the genetic lineages of *Ap. astaci* recorded in Ireland (see Supplementary Information for details). Genotyping using microsatellites [33] and haplotyping using mitochondrial DNA [34] were performed and reported in the 2018/2019 NCPSP report. These were complemented with a qPCR assay [32] in the 2020/2021 NCPSP which identified the A, B, C, and D genotypes in 15 catchments; see Supplementary Materials S2 for further details on these putative genotype results. The genetic lineages of *Ap. astaci* remain to be determined in four infected catchments. Importantly, however, none of these results went through peer review nor were any data made publicly available, making it difficult to scientifically verify the results. In particular, the reporting of the B and C genotypes from a single animal is a significant finding, because genotype C has not been reported in wild European crayfish populations. Moreover, the determination of the pathogen’s genetic lineage based on water derived eDNA is questionable, although possible, and would depend on a sufficient concentration of *Ap. astaci* DNA in the environment. The Di Domenico et al. (2021) assay is a sensitive qPCR-based test but was based on DNA sourced from tissues and not eDNA samples. Moreover, to the best of our knowledge, genotyping is not routinely completed using environmental sources and so these data require further verification. Regarding Northern Ireland, determining the genetic lineages of *Ap. astaci* in the samples present in the region was not possible due to the way the samples were stored (pers. comm). Both Irish and Northern Irish authorities should send anonymised tissue samples to several external expert labs to identify/verify the genetic lineages of *Ap. astaci* present in Ireland.

### 6.5. Lack of Standardisation

The four recently published reports/article analysed in this review span from 2016 to 2021. The initial study, conducted by ATU, incorporated both eDNA testing and traditional survey methods (Table 1). In contrast, the subsequent study report, also by ATU, focused solely on conventional sampling techniques. The 2017 survey omitted eDNA testing, as this was not a requirement specified in the tender (grant) application. Although these two surveys were overseen by different principal investigators, they shared the same sampling team, suggesting a comparable level of effort in data collection.

In comparison, the National Crayfish Plague Surveillance Program (NCPSP) surveys demonstrated a higher degree of standardisation. These surveys predominantly utilised eDNA testing. The assays used, including genotyping, were reported as well-optimised, enhancing the reliability and consistency of the findings. Unfortunately, the protocols and running conditions to determine the genetic lineages presented in the NCPSP reports (presented in Supplementary Materials S2) have not been provided nor peer reviewed. Therefore, those results must be considered as preliminary. The NCPSP traded a more thorough sampling approach for greater coverage and invested more effort collecting samples from a greater number of sites compared to previous surveys. The value of this trade-off is unclear. During the short summer survey period of the 2017 crayfish survey, 127 sites were sampled, which only increased to 168 sites for the annual NCPSP 2021 report period.

### 6.6. Continued Public Engagement

Both Irish and Northern Irish authorities have issued press releases regarding crayfish plague events, and these have been covered by local and national newspapers. However, the topic contains several examples of easily misunderstood or confusing vocabulary. For instance, “crayfish plague” can be misunderstood to be a plague of crayfish (i.e., too many crayfish). Regarding public engagement, the term “voluntary ban” combines two contradictory concepts, is unintuitive, and lacks clarity. Future public engagement campaigns should use plain language, avoid technical jargon, and provide clear and concise descriptions and explanations. Pictures and diagrams could be used to describe complex concepts. Signs should be transparent regarding the challenges of managing crayfish plague. It is vital for the public to grasp the severe impact of crayfish plague on the freshwater environment, emphasising the urgency and significance of this issue.

## 7. Concluding Remark

The spread of crayfish plague in Ireland presents a significant conservation challenge and it is imperative that Irish and Northern Irish authorities adopt a more proactive and comprehensive multi-faceted approach to crayfish and crayfish plague management. Effective management requires not only immediate action in response to outbreaks but also a long-term strategy incorporating both modern and traditional sampling methods, legislative changes, and public awareness campaigns. The future of Ireland’s only crayfish, and the ecosystems they inhabit, hinges on a coordinated and dedicated effort to combat this devastating disease. With greater and concerted efforts, there is hope for the successful conservation and recovery of Ireland’s native crayfish populations and the preservation of their important role in freshwater ecosystems.

## 8. Data Sources

The historical crayfish plague event was documented first-hand by Reynolds [16]. Data from four recent surveys completed in 2016, 2017, 2019, and 2021, were retrieved from their respective articles or reports: Mirimin et al. (2022) [18]; White-clawed Crayfish *Austropotamobius pallipes* survey in designated SACs in 2017 [19]; National Crayfish Plague Surveillance program 2018–2019 [20]; and Report National Crayfish Plague Surveillance Programme 2020–2021 [21], respectively.

## Figures and Tables

**Table 1:** Surveys undertaken to detect the presence of *Aphanomyces astaci* in Ireland between 2015 and 2021.

Report */Article ^#^ Title	Methods	Conducted by	Year/s	Reference
Crayfish Extinctions and Crayfish Plague in Central Ireland ^#^	Conventional survey	Reynolds	1987	Reynolds [16]
Investigation of the first recent crayfish plague outbreak in Ireland and its subsequent spread in the Bruskey River and surrounding areas ^#^	Conventional survey; eDNA	ATU	2016	Mirimin et al. [18]
White-clawed crayfish *Austropotamobius pallipes* survey in designated SACs *	Conventional survey	ATU	2017	Gammell et al. [19]
The National Crayfish Plague Surveillance Program, Ireland *	eDNA	MI for NPWS	2018 2019	White et al. [20]
The National Crayfish Plague Surveillance Programme, Ireland *	Conventional survey; eDNA	MI for NPWS	2020 2021	Swords and Griffin et al. [21]

Year indicates the year the surveys were completed; Atlantic Technological University (ATU), Galway; Marine Institute (MI); National Parks & Wildlife Service (NPWS); * Report of results issued; ^#^ Peer-reviewed article published.

## Data Availability

All data were obtained from open access and available field reports and a peer-reviewed research article.

## Supplementary Materials

The following supporting information can be downloaded at: https://www.mdpi.com/article/10.3390/microorganisms12010102/s1, Table S1: The molecular assays used to determine the genetic lineages of *Aphanomyces astaci* in the National Crayfish Plague Surveillance Program 2018–2021. Figure S1: Distribution of *Aphanomyces astaci* genotypes across infected catchments in Ireland. Table S2. Genotyping data generated from microsatellite markers and qPCR genotyping presented in National Crayfish Plague Surveillance Program 2020/2021. Table S3. Positive sites for *A. astaci* and site details from the 2018–2019 National Crayfish Plague Surveillance Program. Table S4. Positive sites for *A. astaci* and site details from the 2020–2021 National Crayfish Plague Surveillance Program. Figure S2. An advertisement for *Cambarellus patzcuarensis* on DoneDeal in Ireland, September 2023. The advert was online for a minimum of 43 days.
