# Supplementary material for: The Crayfish Plague Pathogen Aphanomyces astaci in Ireland
Source: Microorganisms. 2024 Jan 4;12(1):102. doi: 10.3390/microorganisms12010102 (PMC10819094; doi:10.3390/microorganisms12010102)
Supplement: Supplementary file 1 [file microorganisms-12-00102-s001.zip › microorganisms-2752045-supplementary.pdf]

Supplementary information

The Crayfish Plague Pathogen *Aphanomyces astaci* in Ireland

Daniel J. Brady, Rossa Meade, Julian D. Reynolds, Andreas Vilcinskas and Kathrin Theissinger

S1

Determining the genetic lineages of *Aphanomyces astaci* in Ireland

Determining the specific genetic lineages present in Ireland can provide insights into the pathogen's spread and movement within and among water catchments. To determine these specific genotypes and haplogroups from dead crayfish tissue samples and also environmental (e)DNA from water, a number of techniques were used (Table S1) by the respective authorities of the National Crayfish Plague Surveillance Programme (NCPSP). However, neither the 2018/2019 nor the 2020/2021 NCPSP reports have been peer reviewed. Neither has the data produced to determine genetic lineages been validated nor the raw data and the materials and methods been made public. Although possible with sufficient environmental DNA concentrations, to the best of our knowledge, single gene markers such as microsatellites or mitochondrial DNA are not routinely used to verify the haplogroup or genotype of *Ap. astaci* from eDNA, due to the variable DNA concentrations in the water samples. Therefore, the data presented in the NCPSP reports must be considered preliminary until the data are validated by peer review.

Table S1. The molecular assays used to determine the genetic lineages of *Aphanomyces astaci* in the National Crayfish Plague Surveillance Program 2018-2021.

Method	2018/2019 Report	2020/2021 Report	Data Required for Verification	Provided	Reference
Microsatellite Genotyping	Yes	Yes	Microsatellite primers	No	Grandjean *et al.*, 2014 [33]
			Allele size data	No	
mtDNA Sequencing	Yes	No	Raw sequencing data	No	Makkonen *et al.* 2018 [34]
			Sequence alignments	No	
qPCR Assay [2]	No	Yes	Raw data	No	Di Domenico et al. 2021 [32]

The 2018-2019 NCPSP report used microsatellite genotyping and mtDNA haplotyping to identify genotypes of *Ap. astaci* in various catchments. Microsatellite genotyping are assumed to be based on Grandjean *et al*. 2014 markers and revealed three different genotypes. Mitochondrial DNA sequencing, as described by Makkonen et al. 2018, was employed to determine the haplogroups of *Ap. astaci* present. The report indicated at least two, possibly three, separate introduction events of crayfish plague in Ireland. Important details, like the specific microsatellite markers used or the sequencing reads, were not provided, necessitating a need for further validation and detailed documentation of the methodologies used.

The 2020-2021 NCPSP report used an additional genotyping method to identify *Ap. astaci* genotypes across various catchments, a qPCR genotyping assay described by Di Domenico *et al*. 2021 and "further optimized microsatellite markers" without any specifications provided. The distribution of these preliminary identified genotypes can be seen in Figure S1 and Table S2. The NCPSP identified multiple *Ap. astaci* genotypes, suggesting varied origins and potential

multiple introduction events of crayfish plague in Ireland. The report highlights the importance of consistent genotyping techniques in monitoring and managing crayfish plague, although detailed methodology and peer review of these processes are yet to be confirmed.

The identification of multiple *Ap. astaci* genotypes in Ireland tentatively confirms that the pathogen was introduced multiple times rather than just once. This assumption is supported by the fact that no non-indigenous crayfish species (NICS), the definitive hosts of the pathogen, have ever been found at or near the infection sites. This pattern implies that the pathogen's spread across Ireland has occurred through a series of distinct introduction events, rather than stemming from a single occurrence.

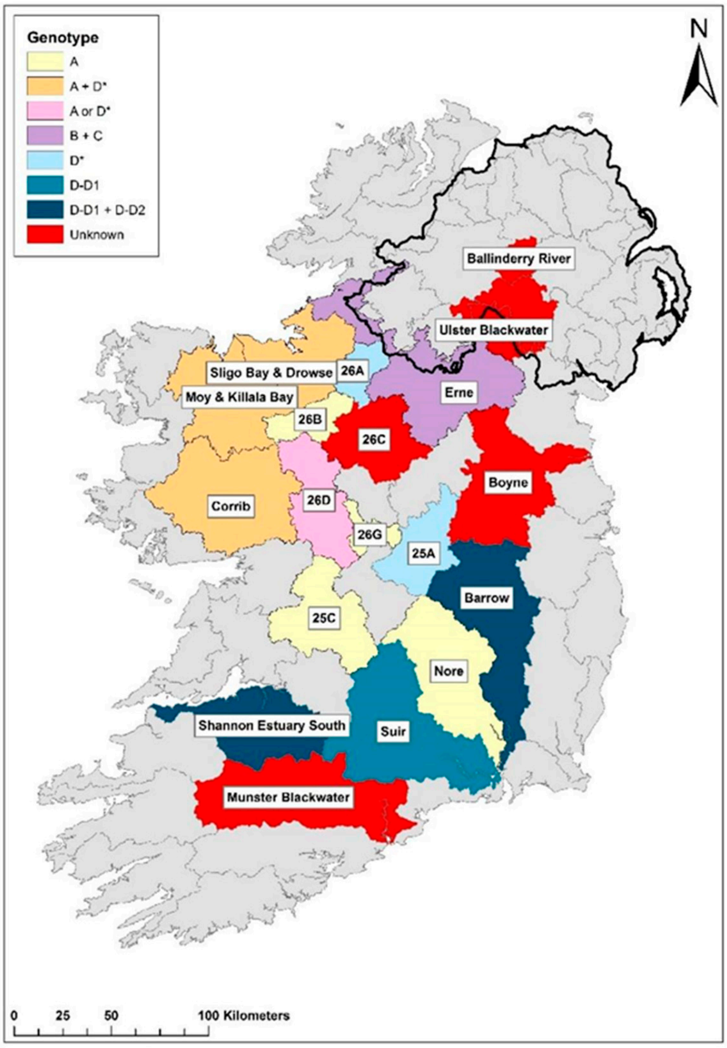

Figure S1. Distribution of *Aphanomyces astaci* genotypes across infected catchments in Ireland. Genotypes presented are reported in the 2021 NCPSP report and were determined by microsatellite markers, mtDNA from the first NCPSP report, and a newer qPCR assay (Di Domenico *et al*., 2021) in the second NCPSP report; black line signifies Northern Ireland. Ballinderry River and Munster Blackwater occurred in 2023 but genotyping details have not been published at time of writing (Nov., 2023).

Table S2. Genotyping data generated from microsatellite markers and qPCR genotyping presented in National Crayfish Plague Surveillance Program 2020/2021.

Catchment	River	Year	Sample type	Genotype
Erne	Bruskey	2015	Tissue	B and C (evidence for both)
Erne	Bruskey	2015	Tissue	B indicated (incomplete data)
Erne	Bruskey	2015	Tissue	B indicated (incomplete data)
Erne	Bruskey	2016	Tissue	B indicated (incomplete data)
Suir	Suir	2017	Tissue	D, D1 haplotype
Shannon 25C	Lorrha	2017	Tissue	A
Shannon ES	Deel	2017	Tissue	D, D2 haplotype
Shannon ES	Maigue	2019	Tissue	D, D1 haplotype
Shannon ES	Maigue	2020	Tissue	D
Shannon ES	Maigue	2021	Tissue	D
Shannon ES	Maigue	2021	Tissue	D
Barrow	Barrow	2017	Tissue	D, D2 haplotype
Barrow	Slate	2019	Tissue	D, D1 haplotype
Corrib	Clare	2018	eDNA	A
Corrib	Clare	2019	eDNA	D
Shannon 26G	Al	2018	Tissue	A
Ulster Blackwater	Ulster	2018	Tissue	Unknown
Ulster Blackwater	Blackwater		Tissue	Unknown
Shannon 26D	Suck	2019	eDNA	A and D (evidence for both)
Shannon 26A	Feorish	2019	eDNA	D
Nore	Nore	2019	Tissue	A
Shannon 26B	Shannon	2019	eDNA	A
Shannon 25A	Clodiagh	2021	Tissue	D
Moy	Moy	2021	eDNA	A and D (evidence for both)
Moy	Moy		eDNA	A and D (evidence for both)
Sligo	Owenmore	2021	eDNA	A and D (evidence for both)
Sligo	Owenmore		eDNA	A and D (evidence for both)

Note that eDNA samples could be unreliable sources of DNA for genotyping.

S2: Data for sites testing positive for *Ap. astaci* reported in the 2018/2019 NCPSP report.

Table S3. Positive sites for *A. astaci* and site details from the 2018-2019 National Crayfish Plague Surveillance Program.

Catchment	Site	Lat	Long	Result
Barrow	Monasterevin Bridge	53.137.830	-7.064.060	positive
Corrib	Claregalway Bridge	53.345.340	-8.942.530	positive
Shannon 26A	Ballyfarnon	54.058.216	-8.194.808	positive
Shannon 26B	Cootehall Bridge	53.981.856	-8.161.176	positive
Shannon 26D	Mount Talbot	53.526.591	-8.284.556	positive
Shannon 26G	River Al Site 1	53.417.440	-7.895.540	positive
Shannon 26G	River Al Site 2	53.412.850	-7.899.800	positive
Shannon 26G	River Al Site 3	53.414.420	-7.902.340	positive
Shannon ES	Adare Bridge	52.568.764	-8.784.244	positive
Shannon ES	Castleroberts Bridge	52.543.670	-8.767.030	positive
Suir	Cahir Bridge	52.374.400	-7.927.130	positive

S3: Data for sites testing positive for *Ap. astaci* reported in the 2020/2021 NCPSP report.

Table S4. Positive sites for *A. astaci* and site details from the 2020-2021 National Crayfish Plague Surveillance Program.

Catchment	Sampling site	River or lake	Sample source	Date	Latitude	Longitude	*Ap. astaci*	Mean Ct
Barrow	Two Mile Bridge	Barrow	eDNA	07/07/2020	53.15546	-7.3692	positive	35.98
Barrow	Owenass	Owenass	eDNA	07/07/2020	53.09624	-7.37968	positive	30.89
Barrow	Two Mile Bridge	Barrow	eDNA	16/11/2020	53.15546	-7.3692	positive	35.93
Barrow	Millgrove	Figile	eDNA	16/11/2020	53.21849	-7.10309	positive	36.98
Barrow	Monasterevin	Barrow	eDNA	17/11/2020	53.13728	-7.06422	positive	36.23
Barrow	Leighlinbridge	Barrow	eDNA	17/11/2020	52.73619	-6.97886	positive	36.2
Corrib	D/S Corrofin	Clare	eDNA	06/08/2020	53.4106	-8.8798	positive	36.7
Moy	Cloonacannana	Moy	eDNA	26/08/2020	53.9674	-8.9303	positive	35.82
Moy	Upstream POS 2020	Moy	eDNA	01/10/2021	54.03657	-8.81681	positive	34.8
Moy	Cloonacannona	Moy	eDNA	01/10/2021	53.96741	-8.93039	positive	35.74
Moy	D/S positive 2020	Moy	eDNA	01/10/2021	53.93793	-9.1034	positive	35.62
Nore	Newbridge Cloncough	Nore	eDNA	22/07/2020	52.942	-7.4442	positive	37.94
Nore	Three Castles bridge	Nore	eDNA	23/07/2020	52.7136	-7.3228	positive	38.57
Nore	Jenkinstown Park	Dinin	eDNA	23/07/2020	52.71489	-7.29203	positive	37.19
Shannon 26B	Boyle Footbridge	Boyle River	eDNA	27/08/2021	53.97244	-8.30143	positive	33.3
Shannon 26C	Drumsna	Shannon	eDNA	16/09/2021	53.92499	-8.01092	positive	37.03
Shannon 26C	Rinn Marina	Rinn River	eDNA	16/09/2021	53.79801	-7.8745	positive	38.01
Shannon Estuary South	Askeaton Main Street	Deel	eDNA	30/06/2020	52.6008	-8.9739	positive	36.06
Shannon Estuary South	Athlacca (Howardstown)	Morning Star	eDNA	01/07/2020	52.4518	-8.6721	positive	26.73
Shannon Estuary South	River Camogue, Manister	Camogue	eDNA	01/07/2020	52.5179	-8.6658	positive	27.06
Shannon Estuary South	Croom	Maigue	eDNA	01/07/2020	52.5189	-8.7183	positive	33.8
Shannon Estuary South	Castleroberts Bridge	Maigue	eDNA	01/07/2020	52.5433	-8.7673	positive	35.08
Shannon Estuary South	Askeaton Main Street	Deel	eDNA	13/11/2020	52.60083	-8.97398	positive	37.27
Shannon Estuary South	River Loobagh	Loobagh	eDNA	12/11/2020	52.39724	-8.66161	positive	37.38
Shannon Estuary South	Bruree	Maigue	eDNA	12/11/2020	52.42356	-8.66107	positive	35.17
Shannon Estuary South	Athlacca (Howardstown)	Morning Star	eDNA	12/11/2020	52.45174	-8.672	positive	32.63
Shannon Estuary South	River Camogue, Manister	Camogue	eDNA	12/11/2020	52.51785	-8.66588	positive	29.71
Shannon Estuary South	Croom	Maigue	eDNA	13/11/2020	52.51892	-8.7183	positive	31.38
Shannon Estuary South	Castleroberts Bridge	Maigue	eDNA	13/11/2020	52.54331	-8.7676	positive	31.39
Shannon Estuary South	Adare	Maigue	eDNA	13/11/2020	52.5687	-8.7839	positive	32.83
Shannon Estuary South	River Loobagh	Loobagh	eDNA	30/06/2021	52.3938	-8.6439	positive	38.94
Shannon Estuary South	Ballinahinch Bridge	Morning Star	eDNA & tissue	09/07/2021	52.40913	-8.43936	positive	30.18
Shannon Estuary South	River Loobagh	Loobagh	eDNA	10/11/2021	52.39724	-8.66161	positive	34.3
Shannon Estuary South	Bruree	Maigue	eDNA	10/11/2021	52.42356	-8.66107	positive	36.12
Shannon Estuary South	Croom	Maigue	eDNA	10/11/2021	52.51892	-8.7183	positive	38.39
Shannon Estuary South	Adare	Maigue	eDNA	10/11/2021	52.5687	-8.7839	positive	37
Sligo	Gurteen	Owenmore	eDNA	10/09/2020	53.9998	-8.5031	positive	34.8
Sligo	Boyle Road, Gurteen	Owenmore	eDNA	24/09/2021	53.98731	-8.48662	positive	30.95
Sligo	Gurteen	Owenmore	eDNA	24/09/2021	53.99971	-8.50383	positive	29.97
Sligo	Gurteen 2	Owenmore	eDNA	24/09/2021	54.01165	-8.49266	positive	31.73
Suir	Ballygriffin	Multeen	eDNA	29/07/2020	52.5153	-7.9917	positive	36.19

Mean Ct shown, for positive result, minimum of 2 replicates must be positive to call positive.

S4: Advertisement of NICS in Ireland.

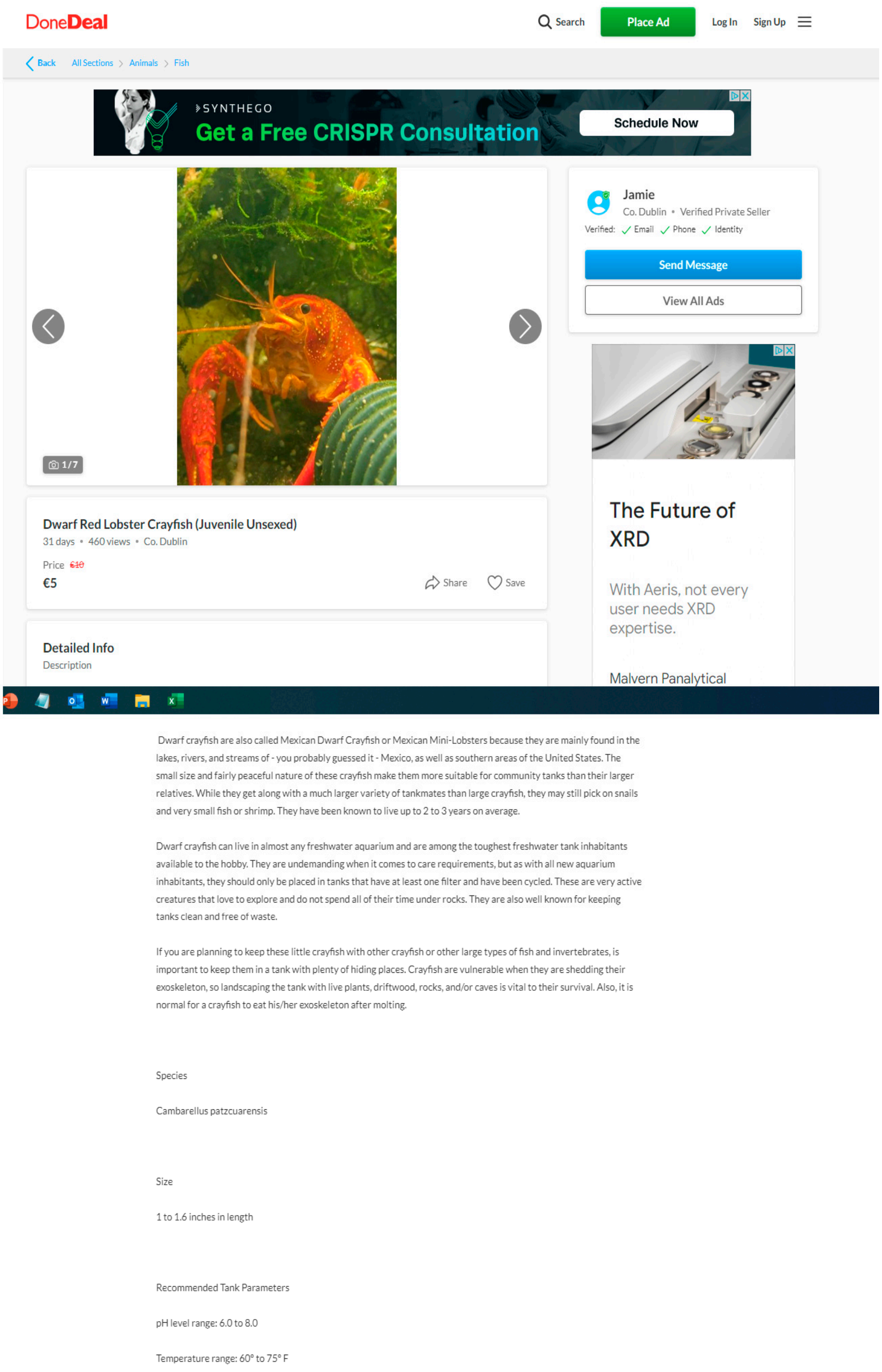

Figure S2. An advertisement for *Cambarellus patzcuarensis* on DoneDeal in Ireland, September 2023. The advert was online for a minimum of 43 days.

References

2. Jussila, J.; Edsman, L.; Maguire, I.; Diéguez-Uribeondo, J.; Theissinger, K. Money kills native ecosystems: European crayfish as an example. Front. Ecol. Evol. 2021, 9, 648495.
32. Di Domenico, M.; Curini, V.; Caprioli, R.; Giansante, C.; Mrugała, A.; Mojžišová, M.; Cammà, C.; Petrusek, A. Real-Time PCR assays for rapid identification of common Aphanomyces astaci genotypes. Front. Ecol. Evol. 2021, 9, 597585.
33. Grandjean, F.; Vrålstad, T.; Dieguez-Uribeondo, J.; Jeli´c, M.; Mangombi, J.; Delaunay, C.; Filipova, L.; Rezinciuc, S.; Kozubikova-Balcarova, E.; Guyonnet, D.; et al. Microsatellite markers for direct genotyping of the crayfish plague pathogen Aphanomyces astaci (Oomycetes) from infected host tissues. Vet. Microbiol. 2014, 170, 317–324.
34. Makkonen, J.; Jussila, J.; Panteleit, J.; Keller, N.; Schrimpf, A.; Theissinger, K.; Kortet, R.; Laura, M.; Sandoval-Sierra, J.; Diéguez-Uribeondo, J.; et al. MtDNA allows the sensitive detection and haplotyping of the crayfish plague disease agent Aphanomyces astaci showing clues about its origin and migration. Parasitology 2018, 145, 1–9.
